# Interference and Shaping in Sensorimotor Adaptations with Rewards

**DOI:** 10.1371/journal.pcbi.1003377

**Published:** 2014-01-09

**Authors:** Ran Darshan, Arthur Leblois, David Hansel

**Affiliations:** 1 Edmond and Lily Safra Center for Brain Sciences, The Hebrew University, Jerusalem, Israel; 2 Laboratoire de Neurophysique et Physiologie UMR8119-CNRS Université René Descartes, Paris, France; 3 Interdisciplinary Center for Neural Computation, The Hebrew University, Jerusalem, Israel; 4 The Alexander Silberman Institute of Life Sciences, The Hebrew University of Jerusalem, Israel; University of Pittsburgh, United States of America

## Abstract

When a perturbation is applied in a sensorimotor transformation task, subjects can adapt and maintain performance by either relying on sensory feedback, or, in the absence of such feedback, on information provided by rewards. For example, in a classical rotation task where movement endpoints must be rotated to reach a fixed target, human subjects can successfully adapt their reaching movements solely on the basis of binary rewards, although this proves much more difficult than with visual feedback. Here, we investigate such a reward-driven sensorimotor adaptation process in a minimal computational model of the task. The key assumption of the model is that synaptic plasticity is gated by the reward. We study how the learning dynamics depend on the target size, the movement variability, the rotation angle and the number of targets. We show that when the movement is perturbed for multiple targets, the adaptation process for the different targets can interfere destructively or constructively depending on the similarities between the sensory stimuli (the targets) and the overlap in their neuronal representations. Destructive interferences can result in a drastic slowdown of the adaptation. As a result of interference, the time to adapt varies non-linearly with the number of targets. Our analysis shows that these interferences are weaker if the reward varies smoothly with the subject's performance instead of being binary. We demonstrate how shaping the reward or shaping the task can accelerate the adaptation dramatically by reducing the destructive interferences. We argue that experimentally investigating the dynamics of reward-driven sensorimotor adaptation for more than one sensory stimulus can shed light on the underlying learning rules.

## Author Summary

The brain has a robust ability to adapt to external perturbations imposed on acquired sensorimotor transformations. Here, we used a mathematical model to investigate the reward-based component in sensorimotor adaptations. We show that the shape of the delivered reward signal, which in experiments is usually binary to indicate success or failure, affects the adaptation dynamics. We demonstrate how the ability to adapt to perturbations by relying solely on binary rewards depends on motor variability, size of perturbation and the threshold for delivering the reward. When adapting motor responses to multiple sensory stimuli simultaneously, on-line interferences between the motor performance in response to the different stimuli occur as a result of the overlap in the neural representation of the sensory stimuli, as well as the physical distance between them. Adaptation may be extremely slow when perturbations are induced to a few stimuli that are physically different from each other because of destructive interferences. When intermediate stimuli are introduced, the physical distance between neighbor stimuli is reduced, and constructive interferences can emerge, resulting in faster adaptation. Remarkably, adaptation to a widespread sensorimotor perturbation is accelerated by increasing the number of sensory stimuli during training, i.e. learning is faster if one learns more.

## Introduction

Transformations that map sensory inputs to motor commands are referred to as sensorimotor mappings [1]. While sensorimotor mappings are already formed at early stages of development [2], they are subject to modifications, since the brain, the body and/or the environment are constantly changing. Plasticity in sensorimotor mappings has been extensively studied in situations where subjects receive sensory feedback during the task, allowing them to correct their motor actions and to adapt to the induced perturbation. These include visuomotor rotation [3], reaching movements under forcefields [4], adaptation in a smooth pursuit eye movements [5], prism adaptation [6], and pitch perturbation in songbirds [7] and in humans [8].

Although these studies involve different sensory modalities and different effectors, they are similar in the sense that they all have sensory goals (targets) and a motor gesture is made to reach the target. They consist of three phases namely a standard phase, in which subjects perform the task under regular conditions followed by an adaptation phase, where subjects perform the same task under the perturbed condition and a washout phase during which the perturbation is removed, and the subject readapts toward baseline. Remarkably, in all these three phases, movements display substantial trial to trial variability. Recent theoretical as well as experimental studies suggested that this variability plays a crucial role in sensorimotor learning and adaptation processes [9]–[11].

Another issue concerns the ability of subjects to generalize the adaptation from one context condition to a different context. This has been investigated by testing how subjects perform upon presentation of sensory stimuli that were not present during the adaptation phase [12], [13]. Generalization is usually good for sensory stimuli that are similar to the one used during adaptation and degrades as the sensory stimuli become different [3], [14]. Remarkably, subjects can even perform worse than in baseline (negative generalization) for sensory stimuli which are very different from those which was presented to the subject during adaptation. This has been observed, for instance, in motor reaching tasks, when the tested stimulus is presented in a direction which is opposite to the adapted direction [4], [14].

The above mentioned studies implicitly assumed that the neural mechanisms for adaptation are driven by a sensory feedback, which supplies a continuous error signal to the subject. Yet, recent studies show that adaptation is possible even without any sensory feedback, when only a binary reward that informs on a success or a failure of a trial is provided to the subject [15]–[17]. Moreover, recent experimental works suggest that reward based mechanisms also affect the adaptation dynamics in sensorimotor tasks even when a sensory feedback is available [18], [19].

However, and not surprisingly, adaptation relying solely on rewards at the end of a trial is more difficult than when a sensory feedback on the performance is provided continuously during the task, as adapting with sensory feedback conveys more information regarding errors. For instance, when visual feedback is available in visuomotor rotation tasks, subjects adapt to large perturbation (e.g. 30 degrees) in a few dozen trials [3], [20], while in the absence of such feedback, but with binary (success or a failure) reward feedback, subjects find it notoriously difficult to adapt. Recent studies, nevertheless, have shown that it is possible to adapt to large perturbations relying solely on rewards if the size of the perturbation is slowly increased between rewarded blocks of trials [17], [21]. The fact that progressively increasing the amount of perturbation makes it possible to adapt, even when the perturbation is large, is reminiscent of the classical shaping strategy [22]. In shaping, the difficulty of the task is increased gradually in order to accelerate learning, or to even make it possible. Although shaping is routinely used in laboratories when training animals to perform complex sensorimotor and cognitive tasks [23]–[25], it is only in recent years that it started to be explored in a theoretical framework [26]–[28].

What neural mechanisms could be involved in this reward based learning? Recent experimental evidence [29]–[31] indicates that rewards modulate local synaptic plasticity via global neuromodulatory signals, e.g. dopamine. When combined with the popular idea that synapses are modified according to Hebbian rules, this leads to the hypothesis that reward signals interact with local neuronal activity to modulate synaptic efficacies [32], [33]. This theoretical paper aims to provide qualitative as well as quantitative insights into the conditions in which sensorimotor adaptation relying solely on rewards can take place. More specifically, we assume that a local learning rule based on the coactivation of pre and postsynaptic neurons is gated by a binary reward signal is the neural basis for modifications of synaptic efficacies [32], [34], [35].

We focus here on adaptation to a rotation during reaching movements where subjects are asked to move a cursor on a screen to bring it within a circular target while the cursor trajectory is rotated (perturbed) by some angle with respect to the hand trajectory. These perturbation tasks are classically used in behavioral studies of sensorimotor adaptation [3]. We consider a simplified network model of this task where adaptation relies solely on binary rewards [17]. The simplicity of the model allows us to analytically study several aspects of the adaptation dynamics. Combining these results with numerical simulations enables us to investigate the ways in which the learning dynamics depend on the model parameters. The key question is how the dynamics of adaptation are affected when the task involves multiple targets. Four main findings are reported: interferences can occur when adapting to multiple stimuli, interferences can slow down the adaptation dynamics dramatically, this depends on the (binary, stochastic) reward, and the slow down can be overcome by using shaping strategies.

## Results

**Figure 1:**
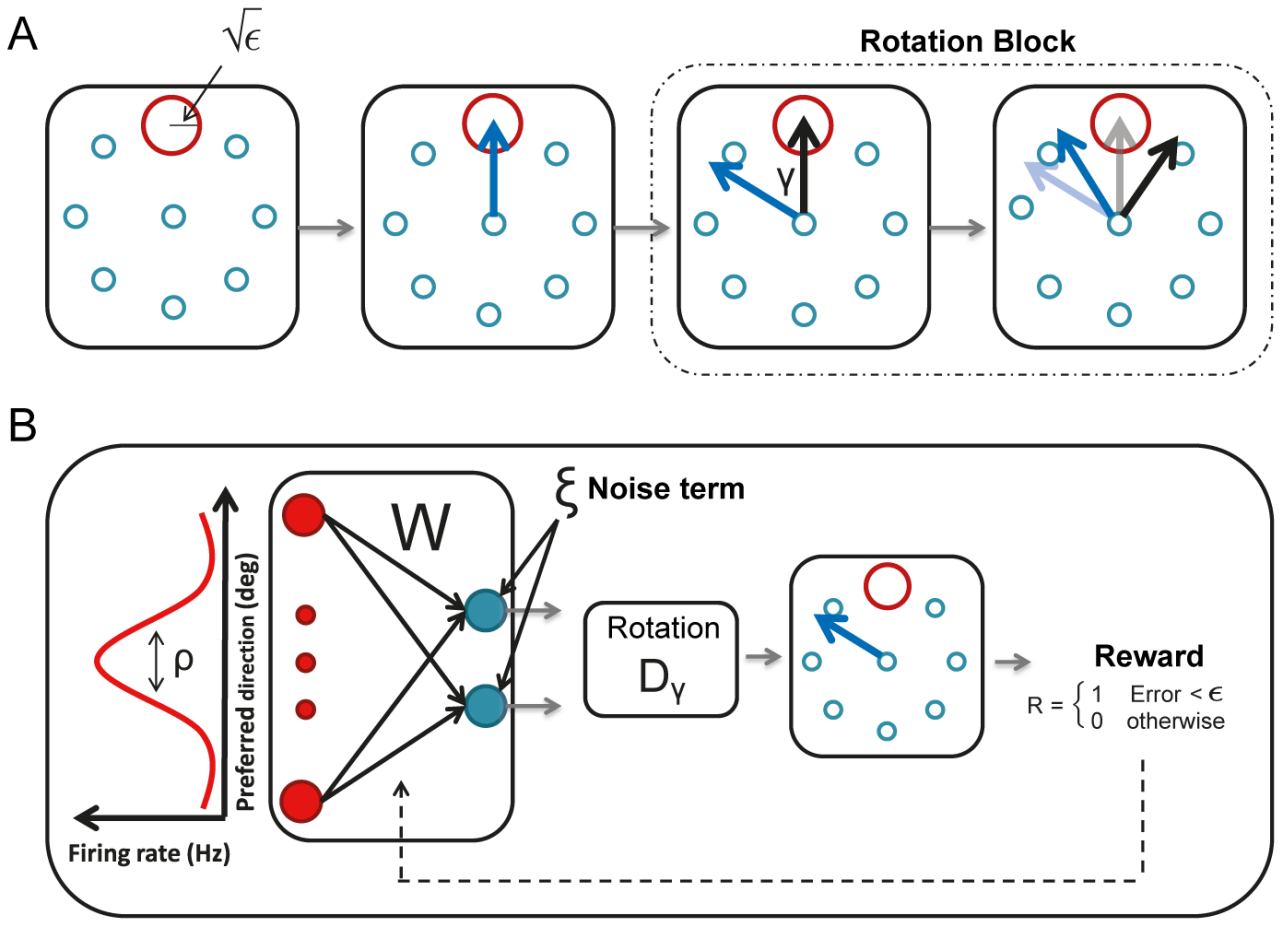
Schematic description of the sensorimotor adaptation task and the model. **A.** The rotation task. From left to right: 1) A circular target (red circle) of radius 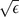 appears on the screen at direction 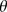 (here 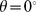) to instruct the subject where to move the cursor. 2) The subject moves the cursor, which is invisible to him, toward the target (blue arrow). The only information available to the subject on his performance is the reward, delivered only if the cursor falls within the target. 3) A perturbation is introduced: the cursor is rotated by an angle *γ* with respect to the direction of the subject's hand movement (black arrow). 4) A learning phase follows where the subject progressively adapts to the perturbation, reducing the distance between the cursor endpoint and the target. **B.** Schematic description of the model. When the target appears, the activity profile of the input layer (red neurons) peaks around the target direction. The parameter *ρ* controls the width of the activity profile. The connectivity matrix between the input and the output (blue neurons) layers is denoted by 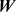. A Gaussian noise with zero mean and a standard deviation of 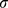 is added to the output layer of the network. The two-dimensional output vector rotated by the matrix 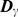 represents the cursor endpoint. A reward is delivered if the distance between the cursor endpoint and the center of the target is smaller than 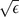. The connectivity matrix 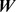 is then changed according to a reward-modulated plasticity rule (see Eq(8)).

We consider the classical rotation experiment [3] in which a subject has to move a cursor on a screen to bring it within a circular target with a radius of 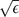; see Figure 1A. At the beginning of the experiment there is no discrepancy between the movement of the hand and the movement of the cursor. We assume that the subject is able to generate the appropriate hand movement to perform the task correctly. A perturbation is then introduced, so that the cursor trajectory is rotated by an angle *γ* with respect to the hand trajectory. The subject has to adapt his movements to this new condition.

In the present work, we focus on the case where the subject receives no visual feedback about the trajectory of the cursor. The only information on performance is a reward provided by the experimentalist at the end of a trial, according to the location of the cursor with respect to the desired target.

Our simplified model for a network which generates the reaching movement is depicted in Figure 1B. Its input layer consists of sensory neurons tuned to the location of the target. It has the geometry of a ring: the preferred direction (between 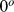 and 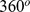) of a neuron corresponds to its location on the ring (see Eq(2)). Hence, when a target appears, the population activity profile in the input layer peaks around a location which is also the target direction. For simplicity we assume that the tuning curves of all the neurons have the same shape. Therefore, the shapes of the population activity profile and the tuning curves are identical. In particular, the tuning width, 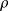, is also the width of the activity profile.

The output layer consists of two linear units. Their activity encodes the 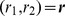 coordinates of the endpoint of the hand movement in the two dimensional environment. The connectivity matrix implementing the sensorimotor mapping between the input and the output layer is denoted by 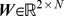. In addition to their feedfoward inputs from the first layer, the output units also receive a Gaussian noise, 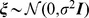 (see Eq(4)), where 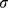 is the 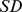 of the noise (also referred to hereafter as the *noise level*). The vector representing the endpoint of the cursor is obtained by rotating the output vector of the second layer, 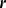, by an angle 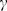 (2×2 rotation matrix- 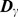).

The reward, *R*, delivered at the end of the movement, depends on the distance between the cursor and the target. Unless specified otherwise it is binary: 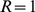 for a successful trial, *i.e.* if the squared distance is smaller than the target size, and 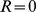, otherwise. The target size is controlled by the parameter 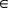 and therefore 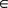 is referred to as the target size in the text.

Following trial *t*, the network adapts to the rotation by modifying the connectivity matrix, 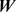, according to the reward-gated synaptic plasticity rule [32], [36]–[38]:
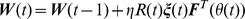
where *η* is the learning rate, 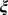 is the noise in the output layer and 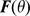 is the activity of the input layer in response to the presentation of a target in direction 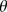. We will assume that the initial value of the connectivity matrix is such that without noise, the network performs the task perfectly for all target directions when 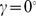 (See Eq(9)). More details about the model are given in Materials and Methods.

The simplicity of the model allows for analytical calculations in the limit of small targets and a better understanding of the learning dynamics. However, the results reported here are grounded on the assumption of a reward-modulated learning rule and are qualitatively independent of the simplifying assumptions used to construct the model. For instance, as shown in Figure S2, the results still hold qualitatively in a more complicated network architecture with a different decoding scheme.

### The learning dynamics for one target

**Figure 2:**
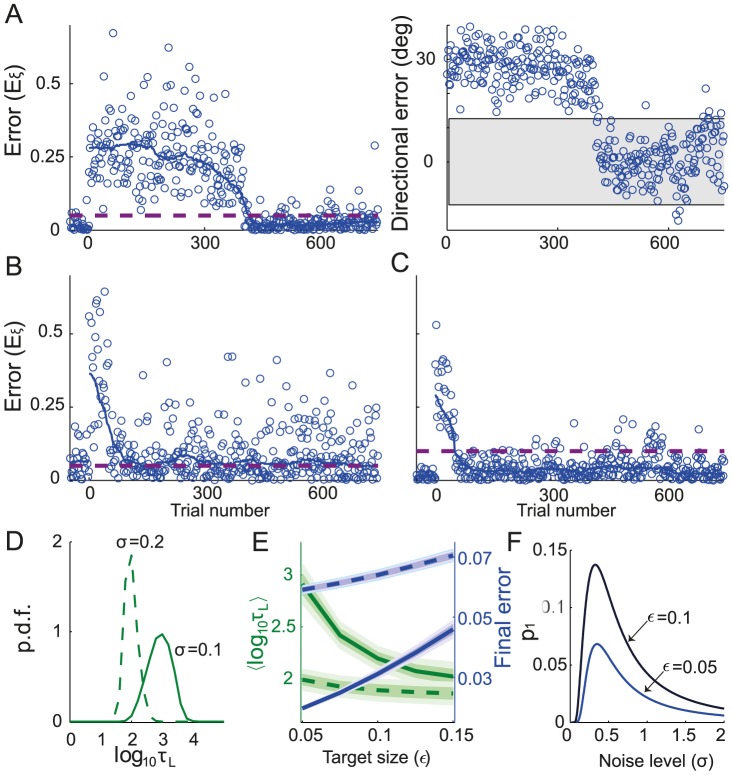
Learning dynamics when the network adapts to the rotation for one target. **A.** An examples of a learning curve for 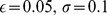. Left: the error is calculated as the squared distance between the cursor endpoint and the target (see Eq. (5)) and plotted as a function of the trial number. The rotation perturbation is applied on trials following t = 0. For display purposes, only one in four trials is displayed. The solid line represents the error, smoothed with a 100 trials sliding median window. Final error of 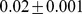 (mean± SE, computed as explained in Materials and Methods). Dashed purple line: Target size. Right: as in left, but only the directional part of the error is plotted against the trial number. The shaded area corresponds to the target size. **B.** Same as in the left panel of **A.** but with 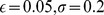 and corresponding final error of 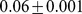. **C.** Same as in the left panel of **A.** but with 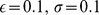 and a corresponding final error of 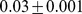. **D.** Probability density function (p.d.f.) of the logarithm of the learning duration. The learning duration (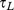) is defined as the number of trials it takes to learn the task (see: Materials and Methods). Target size is 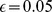. **E.** Trade-off between learning duration and final error. Average of 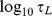 distribution (green) and the final error (blue) are plotted against the target size. The shaded area around the averages corresponds to half SD of the distributions. Solid lines: 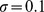. Dashed lines: 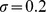. **F.** The probability of getting the first reward, 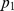 (see Eq. (10)), *vs.* the noise level, 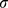 for two values of the target size. In all the panels: 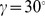.

We first consider the case where the network has to adapt to a rotation of the cursor when only one target is presented. Figure 2A (left) plots the evolution of the error (see Eq.(5)) with the number of trials, hereafter referred to as the learning curve, while the network adapts to an imposed rotation with an angle 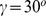. On the right panel we plotted for the same parameters the learning curve of the directional error, which takes into account only the direction of the movement.

The error is large at the beginning of the process and decreases with the number of trials. Importantly, the dynamics strongly depend on the noise. For a low noise level (Figure 2A, 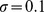), the error remains large for many trials and learning is slow. When the noise level is higher (Figure 2B, 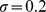) the error declines faster. However, this comes at the cost of increasing the error after learning: the median of this error, called hereafter the *final error* (see Materials and Methods), is larger when the noise level is larger. Similarly, the probability that the network will perform the task successfully, improves more rapidly with the number of trials for 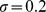 than for 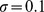, but at very long time it is larger in the latter (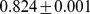) than in the former (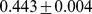) case.

The learning curves plotted in Figure 2A–B were obtained for particular realizations of the noise, 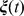. To provide a statistical characterization of these dynamics, we estimated the distributions of the logarithm of the learning duration (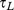) over many realizations of the noise (see Materials and Methods). As shown in Figure 2D, this distribution shifts toward longer learning duration as the noise level decreases.

Figures 2A and 2C plot the learning curves for 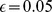 and 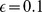 for the same noise level. The learning is substantially faster for 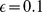 but the final error is larger in this case. This is because when the target size is large, a reward might also be delivered for less precise movement, *i.e.*, for large errors. Figure 2E plots the log learning duration and the final error averaged over 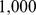 realizations *vs.* the target size: when increasing the target size, the learning duration rapidly decreases, whereas the final error increases.

When the noise level or the target size are increased, the dynamics are typically faster because the probability of generating rewarded trials at the beginning of the learning is larger. As this probability increases, the time for the network to generate a rewarded trial decreases, leading to more updates in the connectivity matrix 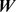; hence the probability of the following trials to be rewarded increases further. This argument can be made more quantitative if one considers how the time to get the first reward depends on 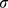 and 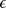. It has a geometrical distribution with a parameter 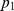 (see Eq.(10)), which is the probability to get the first reward. Lower values of 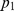 increase the expectation time to the first reward, and thereby the learning duration. When the noise level is low and the initial error is larger than the target size, the network explores a small region of the two dimensional space and the probability of getting a reward is small. In contrast, for very large noise the target is missed most of the time. The probability 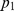 therefore varies non-monotonically with the noise level (Figure 2F). The dependency on target size is simpler: 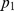 increases monotonically with target size, as it is more likely to reach a larger target.

#### Performance depends on the learning rate parameter

Obviously, the number of trials required to adapt also depends on 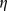, which scales the increment in synaptic strength following a rewarded trial. If the rate is too small, the adaptation will be extremely long, even for large noise or big target size. On the other hand, if this rate is too large learning is likely to be impossible.

**Figure 3:**
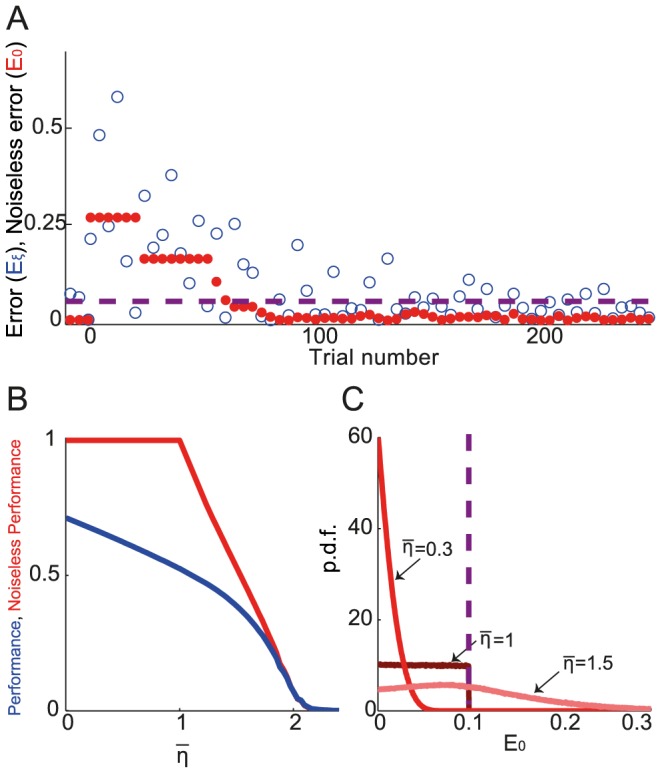
Performance and noiseless performance after learning depends on the learning rate. **A.** An example of the variations of the error (blue) and the noiseless error (red) with the number of trials for 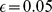 (purple dashed line), 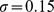 and a normalized learning rate (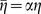, see Eq. (12)) of 0.3. For display purposes, only one in four trials is displayed. **B.** The performance (blue), *i.e.*, the probability that 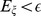 and the noiseless performance (red), *i.e.*, the probability that 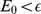 are plotted against the normalized learning rate. These quantities were estimated from simulations of 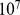 trials, while excluding the transient learning phase. Note that for 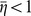 the noiseless performance is perfect. The standard error of the mean is too small to notice. **C.** Distribution of the noiseless error, 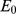, at the end of the learning phase. For 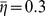, the support of the distribution is bounded by 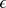. For 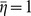, the distribution is uniform for 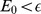 and zero otherwise. For 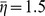 the support of the distribution is bounded but extends beyond 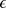. In **B** and **C**: 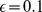; 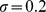.

To analyze how 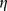 affects the learning of the task it is convenient to decompose the error at trial *t*, 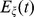, (Eq.(5)) into:
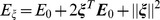
where 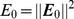 (Eq.(6)) on trial *t* does not depend on the noise, 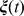 (for more details, see Materials and Methods). We therefore refer to 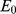 as the *noiseless error*. Changes in the noiseless error are due to updates in the connectivity matrix, 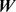, and only occur after rewarded trials. In particular, the noiseless error rarely changes at the beginning of learning, when the probability of getting a reward is low (Figure 3A). The two other terms depend on the noise at trial *t*.

We also define the *noiseless performance* after learning as the probability that the noiseless error will be smaller than the target size at large time. In Figure 3A, the noiseless performance corresponds to the number of trials (red circles) that fall below target size, divided by the number of trials (see also Materials and Methods), when the number of trials is large.

Figure 3B plots the performance (blue) and the noiseless performance (red) for 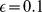 and 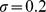 *vs.* the *normalized* learning rate, 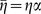 (where 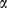 is a constant; see Materials and Methods). The noiseless performance is perfect for 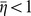. It quickly deteriorates when 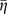 increases beyond 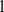, until it becomes extremely small around 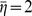. Performance decreases monotonically with 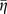 until it reaches 0 around 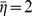. Similar qualitatively results were obtained for other values of 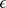 and 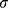 (results not shown).

To better understand how the noiseless performance changes with 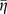, we solved the learning dynamics in the limit of small target size (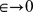) analytically. In this limit, the time between rewarded trials diverges. Using the fact that when a trial *t* is rewarded, the noise, 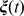, is uniquely determined in this limit, we computed the trajectory of the noiseless error analytically as a function of the number of rewarded trials; see Materials and Methods. In particular, the noiseless error goes to zero for a large number of trials if 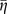 is smaller than 2 and diverges for 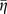 larger than 2.

When 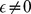 the noiseless error continues to fluctuate with time (as in Figure 3A) in the range (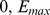), where 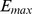 depends on 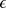 and 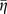. This maximal value can be calculated analytically as shown in Materials and Methods:
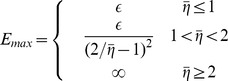
The dependency of noiseless performance with 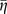 (Figure 3B) stems from this result. When 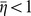 the noiseless error is always smaller than the target size (see example in Figure 3C). Therefore the noiseless performance is always 1. For 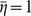 the distribution of the noiseless error, can be calculated analytically (the proof is beyond the scope of this paper). It is uniform in the range (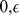) (blue line in Figure 3C). For 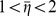 the noiseless error can be larger than the target size (see example in Figure 3C) and noiseless performance is no longer perfect. In fact as 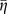 increases, the distribution becomes wider (its SD increases) and noiseless performance decreases monotonously. Finally, when 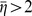 the above equation predicts that the support of the noiseless error distribution is unbounded, and simulations show that it becomes wider; hence the probability of getting a reward is substantially smaller than for 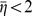.

While noiseless performance is always perfect for 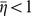, performance can be improved by taking smaller values of 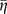 (Figure 3B). This is because the distribution of the noiseless error is sharper when 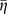 is smaller. However, decreasing 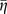 has the obvious consequence of increasing the learning duration. Figure 2 shows that for 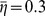 it takes only a few dozen trials to adapt perfectly if the target size is 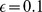. Nevertheless, for smaller 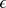 the number of trials increases dramatically. For instance, for 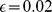 this number becomes extremely large (much larger than 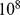) even if 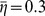.

#### Accelerating the adaptation by shaping the task or the reward

Shaping is a well-known strategy in the context of operant conditioning, which allows a subject to learn difficult tasks in a reasonable amount of time [22]. In shaping strategies, the difficulty of the task is progressively increased. For a given degree of difficulty, the subject has to learn to perform the task, his performance is monitored, and when it is considered sufficiently satisfactory by the experimentalist, the difficulty of the task is increased. A shaping strategy has recently been successfully applied to allow subjects to learn the sensorimotor rotation task relying solely on a reward signal in the absence of visual feedback [17]. In this section, we apply shaping strategies in our model to examine to what extent learning can be facilitated or accelerated.

**Figure 4:**
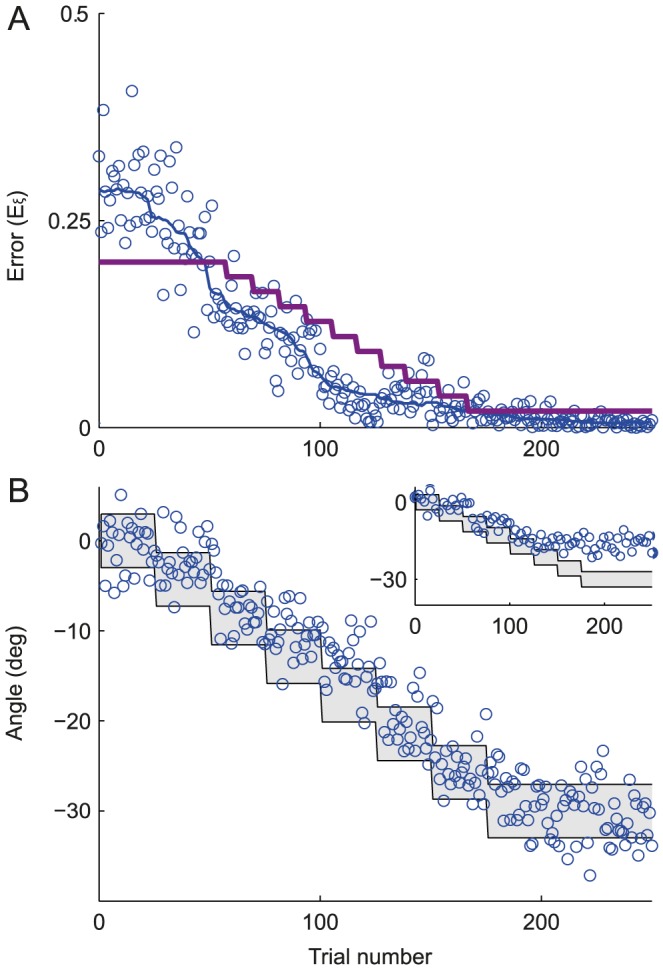
Shaping the task allows the network to adapt to a large rotation angle (here 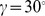) even if the target size and the noise level are extremely small. **A.** Shaping by decreasing the target size, as explained in the text. Parameters: 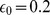; 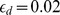; 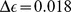; 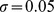. Blue: The error is sampled every 3 trials (dots) and smoothed with a 50 trials median sliding window (line) *vs.* the number of trials. Purple: The size of the target. **B.** Reach angle (in degrees) as a function of the trial number when the rotation angle is progressively increased (see Results). The target size is fixed: 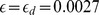. At 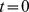, 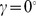. The rotation angle is increased by 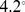 every 25 trials up to 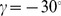. The shaded area corresponds to the target size (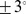 around the target center). Inset: the network is unable to follow the gradual rotation for a different realization of the noise with the same parameters. In both panels: 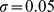.

In the specific case of our sensorimotor adaptation task, the difficulty of the task depends on the target size, the rotation angle and the noise level. For fixed noise level and rotation angle, learning can be shaped by initiating the adaptation process with a large target size and then reducing the size progressively until it becomes as small as desired. This can be implemented as follows. The learning process begins with an initial value of the target size 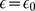, which is large enough for adaptation to be easy and fast. The target size is kept constant, while monitoring the running average of the reward. When the latter approaches a steady state, the target size is decreased by 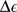 (and the running average of the reward is reinitialized to zero). We repeat this step until the target size reaches the desired value 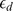. An example of such a shaping strategy is depicted in Figure 4A. Here we plot the learning curve for 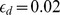, when the adaptation is performed in the presence of very small noise (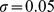), starting with 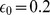. Within fewer than 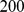 trials the network has adapted and reached a performance of 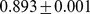. In fact, if the adaptation had been performed with fixed value of 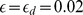, the probability of getting the first reward in fewer than 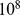 trials would essentially be zero (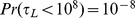), making the network unable to adapt without a tremendous number of trials.

Another example of acceleration by shaping is depicted in Figure 4B. Here, as in Figure 4A, the network has to adapt to a rotation of 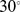. We used similar parameters as in [17] (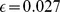, corresponding to a target with a 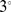 radius and 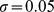). Adaptation is performed using a constant target size, but at the beginning of the learning the angle of the rotation is small and is progressively increased with steps of 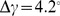 every block of 25 trials. The figure shows that the network adapts in fewer than 200 trials. However, in some of the realizations the network was unable to follow the gradual rotation (see inset). To avoid such cases, one can take smaller rotation steps for longer block of trials, as in [17]. Another possibility is to monitor the running average reward and to change the rotation angle when the latter approaches a steady state, similarly to what we did with the adaptive target size above.

**Figure 5:**
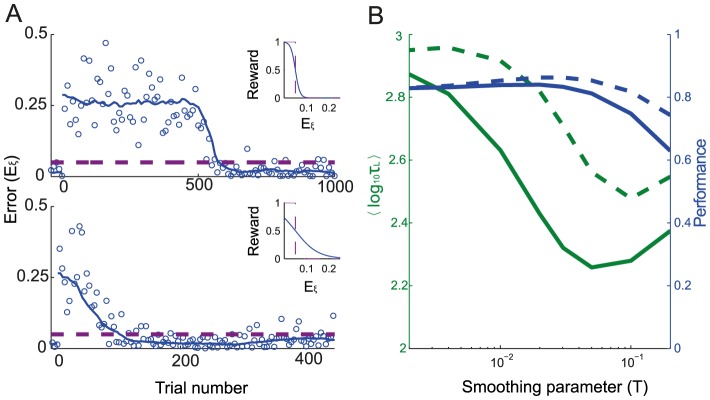
Shaping the reward function accelerates adaptation without impairing performance. **A.** The reward is given by 
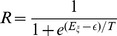
. Top: learning curve for a reward function that changes abruptly around target size (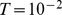). Bottom, main panel: learning curve for a gradual reward function (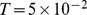). Note the change in the abscissa scale. Inset: The reward function *vs.* the error. The target size is dashed purple line. **B.** The learning duration and the performance *vs.* the smoothing parameter, *T*. Solid lines: Deterministic smooth reward function as in **A**. Dashed lines: Stochastic binary reward delivered with a probability that depends on 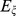 (see Results). In **A** and **B**: 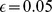; 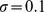.

Binary rewards, as typically used in operant conditioning, provide the subject with a limited amount of information about his performance. For instance, in our model, a binary reward does not convey any information regarding the exact distance between the cursor and the center of the target in case of a miss nor in the case of a success. One way to accelerate adaptation is to shape the reward, *i.e.*, to perform the learning using a reward that depends smoothly on the error. One possibility is to use a deterministic reward given by
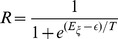
(1)where *T* is a *smoothing parameter*. Figure 5A plots the learning curves for 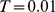 (top panel) and 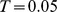 (bottom panel), for fixed values of target size and noise level (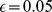, 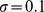). The network improves substantially faster in the latter case than in the former. However, after the error has stabilized, it is comparable in both cases. Figure 5B plots the average logarithm of the learning duration as a function of *T*. It shows that the learning duration increases rapidly for 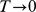, the limit where the reward becomes binary. Note that the learning duration varies non-monotonically with *T* (it is minimum at 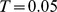). This is because the learning duration also increases for large *T* since a reward which is overly smoothed is less informative.

Remarkably, performance remains very close to 0.8 up to 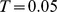. Therefore, using a smooth reward with 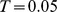 reduces the learning duration substantially without affecting the performance of the network. For *T* above 0.05 performance drops rapidly and the learning duration becomes larger. Hence, in this case 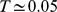 is optimal. We found similar behavior for other values of noise level and target size (not shown).

Another way to provide more information to the subject on his performance, still relying on a binary reward as traditionally used in operant conditioning, is to deliver it stochastically with a probability decreasing smoothly with the error. This can also accelerate adaptation as shown in Figure 5B (dashed lines). Here the reward is a Bernoulli random variable with a parameter 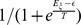.

Altogether, our modeling study predicts that reward shaping strategies, *e.g.*, providing a reward that is a smooth function of the error, as well as other shaping strategies, should be efficient in enabling or accelerating such reward-driven sensorimotor adaptation.

#### Generalization error

**Figure 6:**
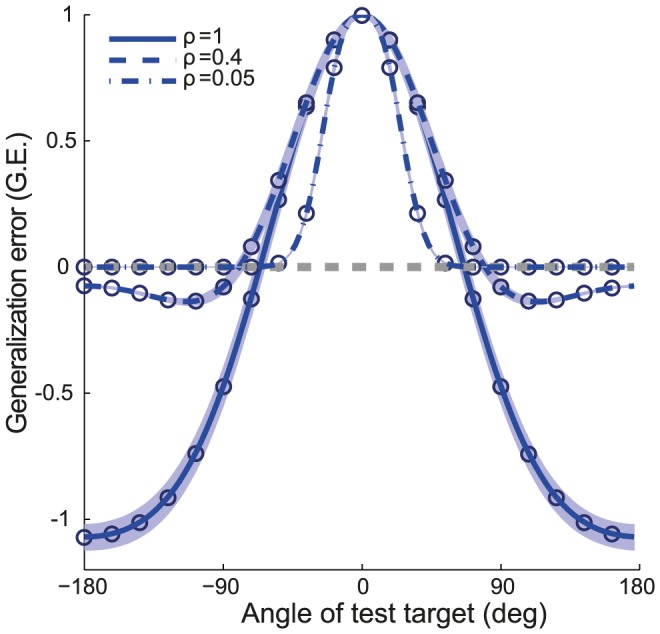
The generalization error (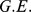) for a new target (defined as the test target), presented after the network has adapted to one target. 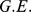
 is plotted as a function of the angle of the test target after adaptation to a target in direction 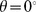. Perfect generalization is when 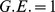. Lines: Analytical result for 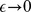 (see Eq.(19)). Circles: Simulation results for 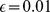. For clarity, the results are displayed for test targets sampled every 15 degrees. The generalization error was averaged over 200 realizations of the noise. Shaded area represents one 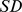 around the averages. Gray line: zero 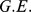. The mapping between 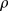 and the half-bandwidth, 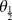, is given in Eq. (3). For instance, 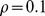 corresponds to 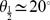 and 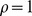 to 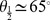. Parameters: 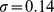; 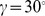.

How does the network generalize the rotation for movement toward targets that were not presented during the adaptation process? To investigate this question we computed the generalization error, 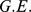 (see Materials and Methods) as a function of the angular distance, 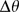, between the target to which the network had adapted and a test target to which it did not adapt. For small target size 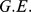 can be calculated analytically (Eq. (20)). Figure 6A plots the results for different widths of the tuning curves, 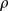. For narrow tuning curves (dashed-dotted line), 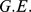 is almost one (*i.e.*, perfect generalization) only when the learned and the test targets are very close. When they are far apart, 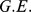 is almost zero. This is because the ability to generalize depends on the overlap, 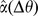 (see Eq. (21)), between the activity profiles in the input layer of the network upon presentation of the learned and test targets. When the tuning curves are narrow, 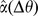 is substantially different from zero only for very close targets and when they are far it is essentially zero. The range in the angular distance in which the generalization error is positive becomes broader when 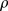 increases (solid line). However, for wide tuning curves, 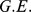 is negative when the targets are far apart. This means that the network performance on far targets deteriorates compared to what it was before adaptation. Note that for intermediate values of 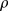 the generalization error can vary non-monotonically with 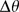 (dashed lines).

The generalization error described here reveals possible interactions between the learning processes for two distinct targets, since adapting for a rotation in one target modifies performance toward others. In what follows, we evaluate the impact of such interactions when adapting the reaching movements to two targets simultaneously and dissect the mechanisms underlying *on-line* positive and negative interactions.

### The learning dynamics for two targets

What is the learning dynamics when the subject has to perform the task for two targets ? How does learning the task for one of the targets affect learning the other one? We addressed these questions in numerical simulations, in which two targets were presented at an angular distance, 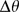, at consecutive times. Similar results were obtained when the targets were presented in a random order with equal probability.

#### Delayed learning

**Figure 7:**
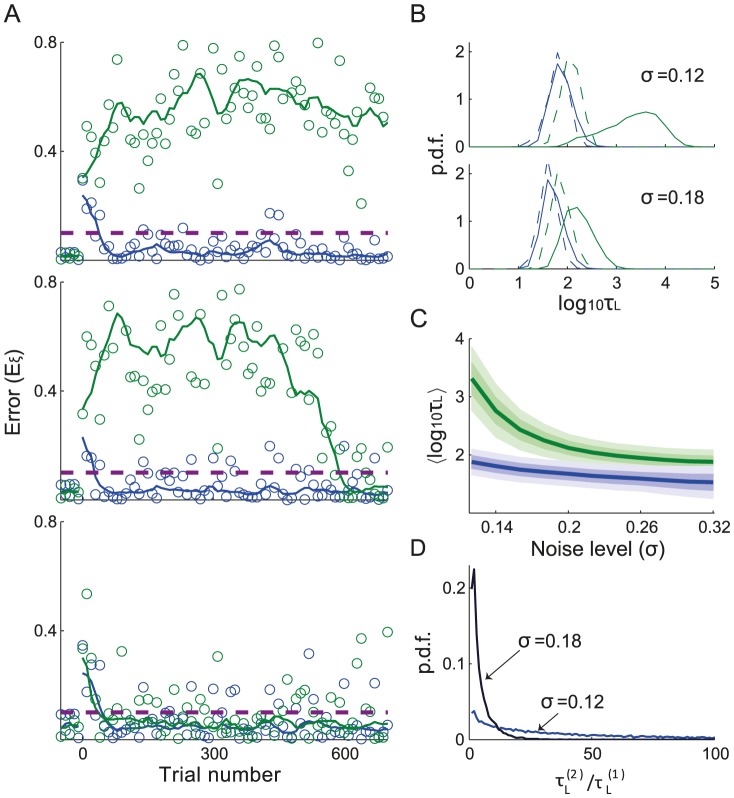
Delayed learning for two targets in opposite directions. **A.** Learning curves plotted against the number of trials for each of the targets, sampled every 10 trials. For the target that is learned first (resp. second) the curve is plotted in blue (resp. green). Top: 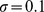. Middle: 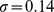. Bottom panel: 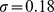. **B.** Distribution of learning duration for two opposite targets for different noise levels. Solid lines: The probability density functions of 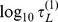 (blue) and 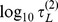 (green) for the two targets (solid lines) where 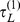 (resp. 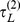) is the learning duration for the target that is learned first (resp. second). Dashed lines: Distributions of 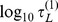 and 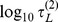 assuming that 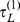 and 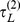 are independent random variables. The distributions were estimated over 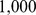 realizations of the noise. Simulations were long enough for the network to eventually adapt to both targets. Top: 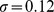. Bottom: 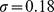. **C.** The average and the SD of the distributions of 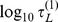
(blue) and 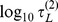 (green) *vs.* the noise level. **D.** The distribution of the ratio 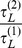 for the two noise level values in **B**.

The top panel of Figure 7A plots an example of the learning curves when the two targets are presented in opposite directions and the noise level is 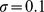. Note that since this noise level and the target size are the same as in the bottom panel of Figure 2A, one might expect that learning the task would be fast. Remarkably, this is not the case here. The error for one of the targets decreases in fewer than 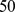 trials, beyond which it keeps fluctuating, most of the time below 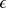. The corresponding performance (see Eq. (26)) is 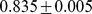. This is in contrast to what happens for the other target, for which the error increases rapidly and keeps fluctuating for the whole duration of the simulation (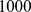 trials) around a mean that is much larger than 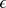. Therefore, in this example, the network is able to adapt in a reasonable amount of time to only one of the targets, in spite of the symmetry of the task with respect to target identity.

Increasing the noise has a dramatic effect, as shown in Figure 7A. For 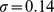 (middle panel), the network is able to learn the task for both targets within 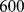 trials, but learning the second target is delayed. We term this effect throughout this paper: *delayed learning*. When increasing the noise level further (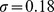), the network adapts almost simultaneously to the two targets (bottom panel).

This effect of the noise in suppressing delayed learning is confirmed in Figure 7B, where the statistics of the logarithm of the learning durations over many realizations of the noise are depicted. The learning duration for the first (resp. the second) learned target is denoted by 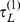 (resp. 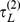). Obviously, the target for which adaptation occurs first depends on the specific realization of the noise. The distribution of 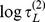 (green) is shifted to the right with respect to the distribution of 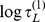 (blue), as for each realization 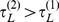, by definition. As a consequence of delayed learning, this shift is larger than would be expected if the task had been learned independently for the two targets (dashed lines). For low noise level this shift is even larger (top panel). Figure 7C shows the averages of the distributions of 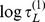 and 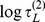 *vs.* 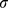. As it was the case for the average of 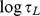 for a single target (Figure 2C), these averages increase for low noise levels. However, the increase is faster for the second target.

The delayed learning effect is also clear in Figure 7D which plots the distribution of the ratio: 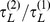, for the same values of 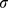 as in Figure 7B. For the highest noise level, in half of the realizations 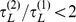. By contrast, for low noise level in more than half of the realizations the learning of the second target is at least 34 times longer than the first one. Overall, delayed learning is reduced when the noise level is increased.

#### Destructive and constructive interference

**Figure 8:**
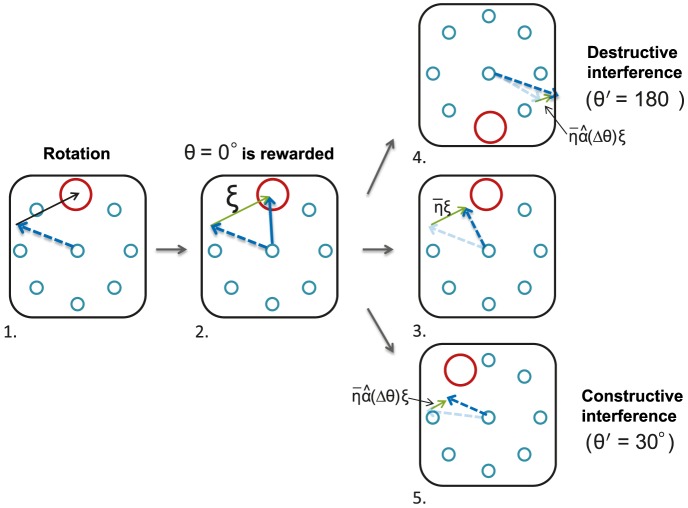
Geometric intuition for the destructive and constructive interferences. Following the perturbation, the cursor is rotated with respect to the output of the network, hence inducing a large noiseless error (black vector in panel 1.). The noise in the output layer (green vector in panel 2) helps the network to explore the 2D environment, until the cursor falls inside one of the targets (panel 2). This trial is rewarded and therefore the connectivity matrix is updated, affecting the output of the network for the next trials. This decreases the noiseless error, for the target for which the trial has been rewarded, as the rotated output of the network is now closer to it (by adding the vector 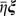, panel 3). This update moves the rotated output *away* from the target in the opposite direction since the vector 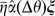 is away from it. This results in an increase in the error, referred to as *destructive interference*. The probability of a rewarded trial for this target is now substantially reduced, delaying learning for that target. A similar effect occurs when the two targets are sufficiently far apart. However, when they are close (panel 5) the interference becomes *constructive*, since after the update of the matrix, the rotated output gets closer to both targets. Note that the overlap, 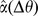, depends on the width of the tuning curves (see Materials and Methods).

This delayed learning can be understood with a geometrical argument, as explained in Figure 8. When the network generates a rewarded trial for one of the targets, it affects the outcome of the second target. Hence, when the targets are in opposite directions, and if the tuning curves are sufficiently broad, this results in an increase in the error of the second target (see also Figure 7A). In other words, the learning processes for the two targets interfere *destructively*. As a result, the probability of generating a rewarded trial for the second target is reduced. Note that according to this argument if the targets are sufficiently close, the interference becomes *constructive*.

**Figure 9:**
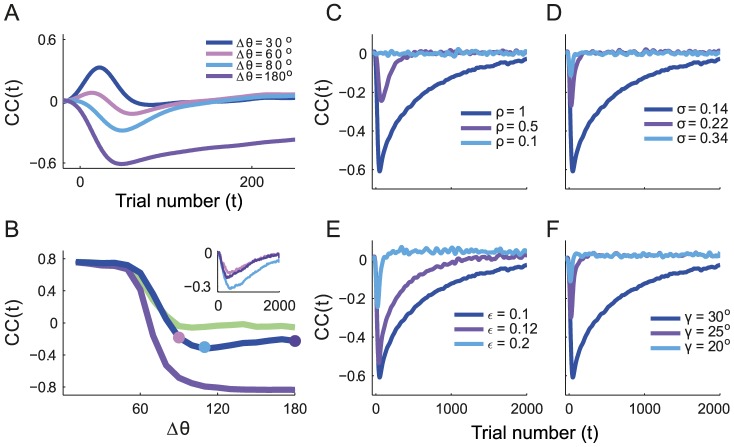
Destructive and constructive interferences are a function of the model parameters. The correlation coefficient, 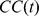, characterizes the strength and the nature of the interference during learning of the rotation task for two targets. **A.** 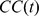 for different values of the angular distance between the targets. The interference becomes constructive when 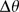 decreases. **B.** The extremum of 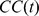 over *t*, 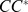, plotted against 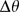 for different values of 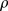. Purple: 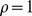. Blue: 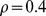. Green: 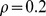. The width of the curve was chosen to correspond to the 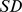 of 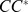, estimated by bootstrap. Note the slight non-monotonicity for 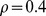. Inset: 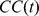 for 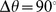, 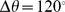, 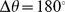 for 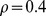 (same color code as for the dots on the main figure in this panel). Parameters: 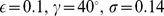. **C–F** 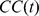 is plotted for different values of 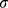 (**C**), 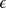 (**D**), 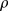 (**E**) and 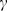 (**F**). In all these figures, 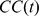 was calculated over 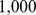 repetitions. The result was low-pass filtered to suppress fast trial-to-trial fluctuations for the sake of clarity. Consequently, there is a causality artifact around 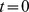 and 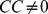, although it should be. The standard errors estimated by bootstrap are small and are not plotted.

To further analyze the interference in adaptation to the two targets, we considered the correlations between the errors at consecutive presentations of the targets. For that purpose, we estimated the time dependent correlation coefficient (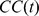) of the errors over different realizations (see *Materials and Methods*). A destructive interference corresponds to negative correlations, whereas a constructive interference corresponds to positive correlations. Figure 9A shows how the sign and the time course of the 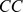 change with the angular distance, 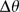. For the first few trials, usually none of the presentations of the targets are rewarded and, therefore, the matrix 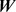 does not change. Hence, during the first trials, 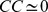. For a sufficiently large number of trials the network adapts to the two targets and 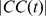 reaches some stationary value.

The results in Figure 9A show that the temporal profiles of 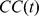 are qualitatively similar for 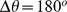 and 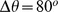, but in the latter case 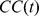 is less negative, indicating a reduction in the destructive interference. By decreasing the angle further to 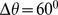 the shape of 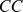 becomes biphasic. In the latter case the nature of the interference changes during adaptation from constructive to destructive. Finally, for sufficiently small 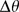, the interference is always constructive. For the parameters in Figure 9A, this is already the case when 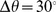.

Figure 9B plots the extremum of 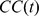, 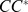, against 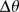, for different widths of the tuning curves. For broad and sharp tuning curves, 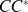 varies monotonously with 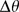 (Figure 9B, purple and green lines). For intermediate degrees of tuning (blue line), 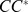 can display non-monotonous variations with 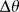 (see also the inset in the figure). In fact, it reveals that the interference can vary non-monotonously with the angular distance, depending on the width of the tuning curves. This non-monotonicity can be grasped from the geometric intuition in Figure 8. The interference is more destructive when 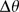 is large; however, as 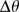 increases, 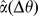 becomes smaller, making the interference less effective. A more rigorous proof is given in Material and Methods.

Similarly, the interference for fixed 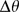 depends on 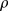 as the overlap, 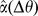, becomes smaller when 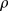 decreases. This is depicted in Figure 9C, where we plot 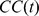 in the case of two targets in opposite directions, for three values of 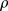. Decreasing the width of the tuning curves results in smaller values of 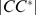. For very sharp tuning curves, interferences are minimal and 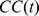 remains very small during the whole learning process. In fact, in the limit 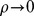, the adaptation process to each of the targets is independent.

Finally, Figure 9D displays 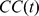 for three values of noise level. The same qualitative behavior is observed in all these cases; however, 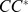 is less negative and 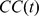 recovers faster when the noise is stronger. This is because increasing the noise decorrelates the adaptation process for the different targets, thus reducing the destructive interference. This is in line with the results displayed in Figure 7.

#### Destructive interferences are reduced by shaping the task or the reward

**Figure 10:**
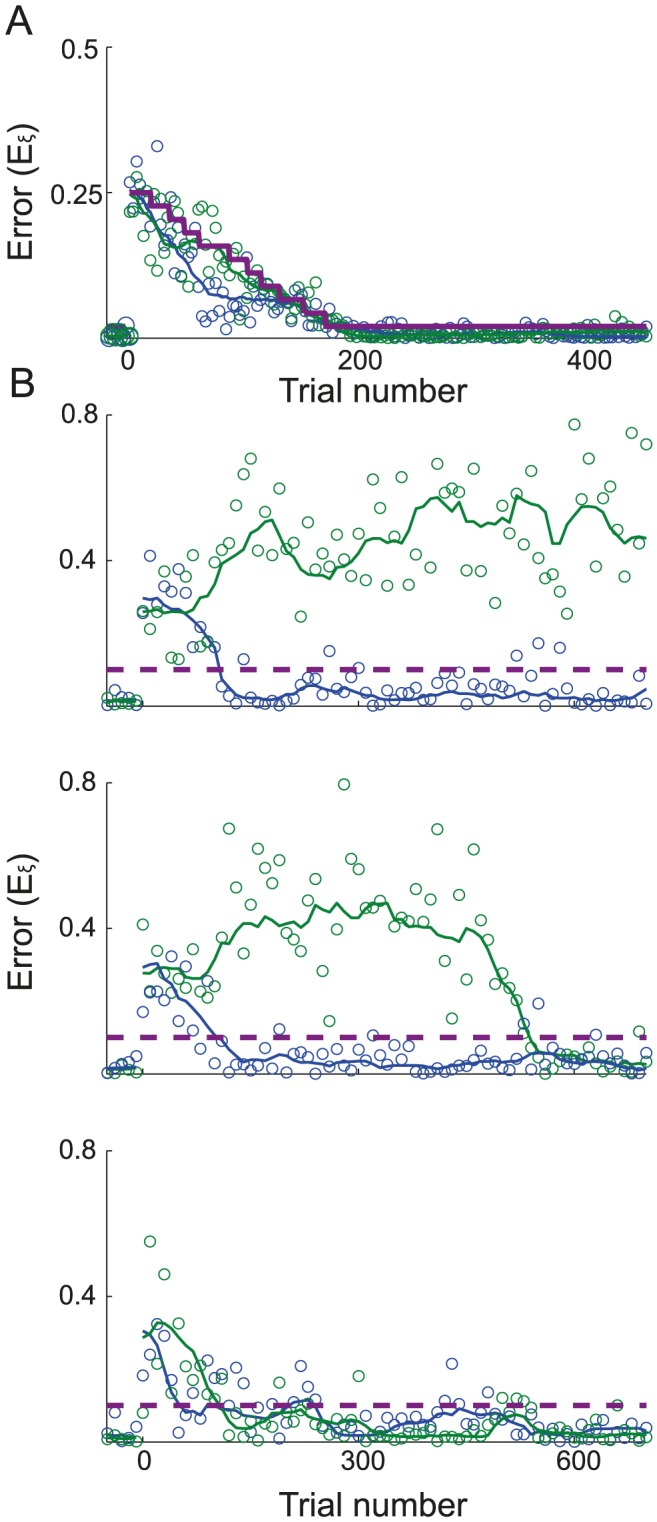
Shaping the task or the reward reduces the delayed learning effect. **A.** Learning curves for two targets in opposite directions. The task is shaped by reducing the target size. Parameters: 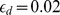; 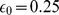; 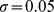. The running averages of the reward were monitored for the two targets separately. When both averages reached a steady state the target size was decreased by 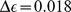. The error was sampled every 3 trials. **B.** Adaptation with a smooth reward function, Eq. (1). Top: 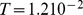. Middle: 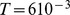. Bottom: 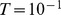. Parameters: 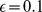; 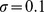. The error was sampled every 10 trials.

Increasing the target size (Figure 9E), as well as reducing the rotation angle (Figure 9F) reduces 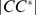, and hence the destructive interference, when adapting for two targets in opposite directions. Therefore, we expect that shaping strategies which gradually manipulate these parameters can help overcome the delayed learning effect. Figure 10 shows that this is indeed the case, when changing the target size adaptively during the adaptation. The running average of the reward signal for each target was monitored *separately* and 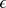 was decreased by 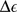 only when both running averages reached a steady state. In this case, the network adapts to both targets quickly and simultaneously. Similarly, shaping the task by increasing the rotation angle progressively reduces the destructive interference and accelerates the learning (data not shown).

Finally, there is less interference if learning is performed with a reward which depends smoothly on the error (Eq. (1)). As depicted in Figure 10B, this results in a suppression in delayed learning. Increasing the smoothing parameter reduces 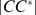. For instance, for the parameters of Figure 10B, 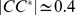 for 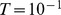, whereas 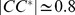 for 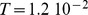. Similar results are found if the reward is binary but stochastic, with a probability that is a function of 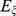 (not shown).

### Learning faster by learning more

How does the learning duration, *i.e.*, the time to learn the task for all the presented targets, vary with the number of targets? We simulated the learning of *m* targets, whose directions were *evenly distributed* between 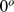 and 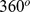. We took a small target size (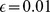), so that up to 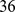 non-overlapping targets could be considered (for targets presented on a circle with radius 1).

**Figure 11:**
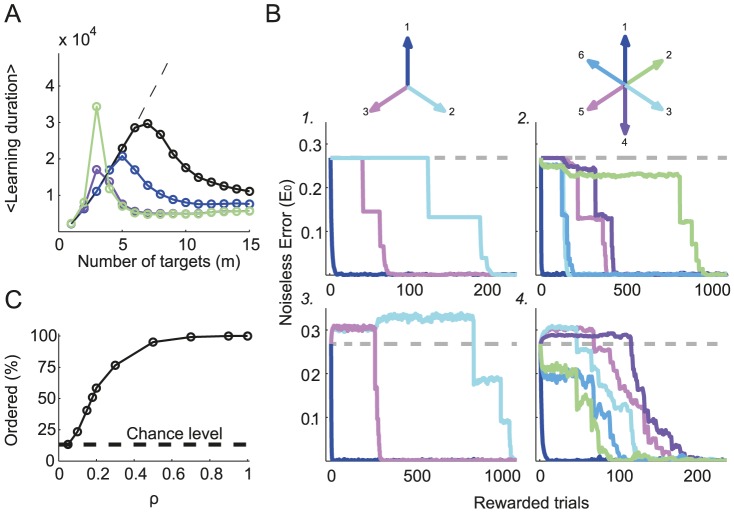
Adaptation to multiple targets. **A.** Average total number of target presentations required to learn the entire task *vs.* the number of presented targets, *m*. The targets are evenly distributed (between 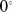 to 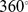). Black: 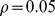. Blue: 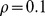. Purple: 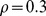. Green: 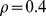. Dashed black line corresponds to learning the targets independently from the p.d.f. of 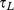, which was estimated from adapting to one target. **B.** Examples of the *noiseless* error during the learning, plotted *vs.* the number of *rewarded* trials. The target direction is color coded. Dashed gray lines: The initial noiseless error for 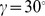. **B.1** and **B.2** are examples of the noiseless error for narrow tuning curves (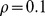) in the case of 3 and 6 targets respectively. The plateau in the noiseless errors indicates that there is no interference between the targets. **B.3** and **B.4** are examples of the noiseless error for wider tuning curves (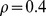) in the case of 3 and 6 targets respectively. The increase in the noiseless error above the initial error for some of the targets is the result of the destructive interference between far targets. **C.** The fraction of ordered realizations when 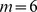 as function of 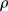. Chance level is 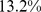. An ordered realization is defined as learning the targets in a close-to-far order, as in the example in **B.4**. The statistics were calculated over 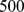 realizations. For all the results presented in this figure: 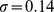.

Figure 11A plots the average time to learn the entire task in terms of the total number of target presentations for a fixed noise level and different values of tuning widths. It shows a non-monotonic dependency with the number of targets. This contrasts the monotonically increasing learning duration when targets are learned independently with the same noise level and target size (dashed line).

#### Narrow tuning curves

When the tuning curves are narrow (black and blue curves) and for small values of *m*, the overlap 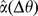 is essentially zero; therefore, there is no interference and the network adapts independently to the different targets. An example is depicted in Figure 11B.1 for 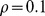. In this figure, the *noiseless error* for all three targets is plotted against the number of *rewarded* trials. Independence is indicated by the fact that abrupt changes in the noiseless error for one of the targets do not affect the noiseless error for the other targets. The overlap only becomes significant when the targets are close enough, resulting in constructive interference (see also Figure 9A). In fact, when *m* increases, the adaptation for close targets interferes constructively, as depicted in Figure 11B.2 for 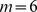. In this example, learning target 1 (see color coding in the figure) does not affect the learning of targets 3, 4 and 5 within the first 200 rewarded trials. It does, however, reduce the noiseless error for the closer targets, *i.e.*, 2 and 6. The constructive interference is also noticeable for the rest of the targets. This constructive interference between close targets facilitates adaptation and explains why the learning duration decreases for larger *m*, and the overall non-monotonicity of the learning duration with *m*.

#### Wide tuning curves

For wider tuning curves, interferences are already present for a small number of targets, but they can be *destructive* when the targets are far apart. For instance, for 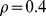 and 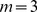, improvements for one target result in an increased noiseless error, above the initial error, for the other targets (Figure 11B.3). However, as in this case 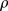 is not too large, adaptation is almost independent with 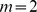 (green curve in Figure 11A). Similar to the case of narrow tuning curves, constructive interference between close targets emerges when *m* is increased. A representative example of adaptation with 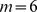 and 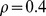 is plotted in Figure 11B.4. Learning target 1 reduces the noiseless error for the two close targets, whereas the error for the other three targets, which are farther apart, becomes larger than their initial values. In this case, constructive interference among the close targets competes with destructive interference between targets that are far apart.

The drop in the learning duration when increasing *m*, both for wide and narrow tuning curves, implies that learning more targets might be faster than learning only a few. For instance, learning 6 targets for 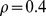 is six times faster than learning only three of them (the 3 that are separated by 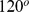).

#### Adaptation is in the close-to-far order when the tuning curves are broad

In Figure 11B.4 (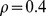) the network learned the task in a specific close-to-far order: after it had learned the first target, it learned the two closest targets (separated by 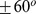), and then the far targets (separated by 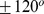 and finally the 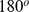 target). Therefore, in this case the targets were learned in an *ordered* way. In contrast, in the example plotted in Figure 11B.2, the tuning curves are narrow (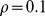) and the learning of the targets is not ordered. This difference stems from the fact that broadening the tuning curves increases the amount of both destructive and constructive interference. As a result, by learning one target, the error of the closer targets is already reduced, whereas learning is delayed for the far targets. Increasing 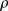 thereby results in more ordered learning. To better characterize how the tuning width controls whether adaptation is ordered or not, we estimated the probability of this occurring as a function of 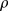. Figure 11C depicts the results for 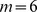. It shows that the fraction of the realizations for which learning is ordered increases monotonically with 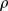.

#### Generalization error for multiple targets

**Figure 12:**
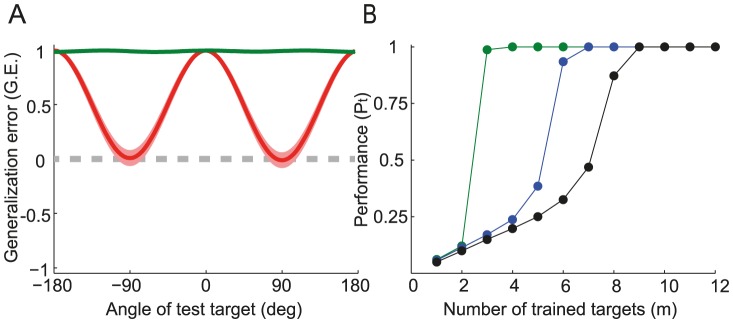
Generalization error (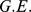) and performance when adapting to multiple targets. **A.** The generalization error *vs.* the location of the test targets, estimated from simulations as in Figure 6. Shaded area represents one 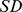 around the averages. Tuning width: 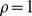. **B.** The noiseless performance (see Eq(25)), averaged over all the tested targets (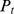) is plotted *vs.* the number of trained targets. See Materials and Methods for details about how this quantity was estimated. Blue: 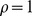. Green: 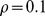. Black: 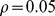. Dashed gray: zero 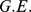 Parameters: 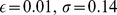.

Figure 12A plots the generalization error after the network has adapted to 2 or 3 targets for 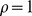. The generalization is essentially one for all tested targets as soon as the network has adapted for three targets (green line). How does the generalization error depend on *m* and 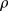
? Figure 12B plots the noiseless performance (see Eq. (25)) averaged over all the test targets (denoted by 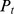), for different values of 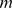 and 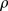. For wide tuning curves, as in Figure 12A, learning the task with only 3 targets is sufficient for almost perfect performance on all the test targets (blue line, 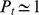). Therefore, there is no added value in adapting to more targets as far as generalization is concerned. However, as explained above, this can substantially accelerates learning. In fact, for the parameters used in Figure 12A the average learning duration is about 170 times shorter for 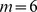 than for 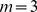. When the tuning curves are narrower, the network only generalizes perfectly to all directions for large 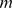 (green and black lines in Fig 12B). Nevertheless, here it is also advantageous for the network to adapt to more targets than required for perfect generalization, since this can accelerate adaptation.

## Discussion

We explored the reward-based component in adaptation processes in a setting in which a subject has to adapt reaching movements to a rotation when the only information available is the location of the target and a binary reward signal indicating success or failure on a trial [17]. The subject thus has to adapt to the perturbation by relying solely on the reward. In the framework of a simplified model of a neural network learning the task, we investigated the ways in which the adaptation dynamics depend on the noise level in the network, the target size, the size of the perturbation and the shape of the reward function. The key finding is that if the network has to adapt simultaneously to several target locations, constructive or destructive interferences between the different movements may occur. Such destructive interferences may result in a severe slowdown in the adaptation process, but this slowdown can be mitigated if the reward changes more gradually from a success to a failure around the target.

If the motor variability is not large enough with respect to the target size and the amount of perturbation (Figure 2), it takes a long time for the network to generate rewarded trials and to update its connectivity matrix. This results in slow adaptation and may be the reason why adaptation in the absence of visual feedback is notoriously difficult for subjects when the rotation angle is too large. For example, at the noise level and target size reported in [17], the probability to generate a rewarded trial in less than 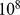 trials for a rotation of 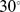 is essentially zero.

The time to adapt also depends on the size of the change in synaptic strength on each rewarded trial; i.e., on the learning rate parameter. We showed that perfect adaptation to one target (i.e. 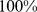 performance in the absence of noise) is possible only when the (normalized) learning rate is smaller than 1. A high learning rate leads to decreased performance and eventually fully impedes adaptation (Figure 3). Therefore, the extent to which adaptation can be accelerated by choosing a large learning rate is limited.

Adaptation is faster for large noise. On the other hand, if the noise is too large, final performance is impaired. Interestingly, motor areas display high variability at the early stages of learning, which becomes smaller afterward. This has been observed in reaching tasks in monkeys [39], as well as in song acquisition in songbirds [40]. Our study suggests that this change in noise level during learning can be functionally important to making a compromise between fast adaptation and good performance.

We showed that when adapting to multiple targets, learning the task for one target can impair performance on other targets due to destructive interference. As a result, the probability that the network will generate a rewarded trial for these targets decreases. Therefore, in this case the same noise level that allows exploration of one movement direction is insufficient when adapting to two or more targets, resulting in a delayed learning effect. Interestingly, when the network starts to adapt to the perturbation to the second target, it does not deteriorate the performance of the network on the first target that was already learned. This is because the network keeps generating rewarded trials for the first target and prevents the connectivity matrix from changing in the wrong direction for the first target.

We also showed that there are cases where the interference that occurs when multiple targets are presented is constructive. In fact, the strength and the nature of the interference depend on the similarities in the distance between the targets (the physical stimuli) and in the overlap of the tuning curves (the neural representations of the stimuli). Adding more targets creates constructive interference and therefore can accelerate adaptation.

### Generality of the results

Models of sensorimotor control and learning frequently assume minimizing a squared error function. This is convenient because of analytical or computational simplicity [13], [14]. However, it was shown that although these models can be a good approximation they tend to penalize large errors excessively [41]. In contrast, we chose to explore adaptation with a binary reward function, as used in experiments. Our results and predictions stem from the shape of the reward function. Specifically, they do not depend qualitatively on the specific choice of the distance error used, but are based primarily on the fact that the reward function varies sharply with the distance to the target center. The dynamics of the adaptation to more than one target depend on the overlap between the tuning curves of the input neurons. However, the precise shape of the tuning curves is not crucial and the results are unchanged if one replaces the Von Mises function we used here with any other tuning curve function, such as a cosine tuning curve (see e.g. Eq. 23).

As a matter of fact, the results we describe are the outcome of the following: 1) the same system is used to learn the task for several targets, leading to interference which depends on the way in which the targets differ physically as well as in their neuronal representation and 2) learning the task for one target can deteriorate performance on another target such that the information provided by the reward when attempting to learn the task for it becomes small, thereby delaying the learning. These two properties of the learning process are not specific to the simple model we investigated here.

In our model, the latter property stems from the fact that the reward varies sharply with the error. The learning rule we used is part of a general family of gradient-like reinforcement learning rules; i.e., learning rules that on average form a gradient ascent on the reward function [35]–[37]. In fact, learning with an on-line Gradient Ascent algorithm with a sigmoidal cost function can result in similar effects (Text S1; Figure S1). It might be claimed that plasticity also occurs when no reward is delivered [42]. Therefore, we also verified that the phenomenology of the model remains qualitatively the same when 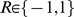 instead of using a 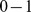 reward function (unpublished data). Note that to avoid a drift of the output vector which occurs when 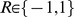, the synaptic weights must be normalized in this case after each trial. Another extension of our model would be to use a reward prediction error instead of an instantaneous reward; e.g., by subtracting a running average of the reward from the instantaneous reward. Delayed learning also occurs with this type of learning rule (results not shown). In fact, previous works have argued that this modification does not affect most of the qualitative behavior of the algorithm [32], [36]. However, it should be noted that in the case of multiple targets, computing the running average of the rewards over all targets is an additional source of interference, as shown recently in [35]. To avoid this, the running average of the reward needs to be monitored for each target *separately*.

We focused on the learning dynamics in a feed-forward network of linear neurons with only two layers. We chose this architecture for the sake of simplicity. However, we verified that similar qualitative behaviors in terms of interference and delayed learning occur in a network model in which an intermediate layer consisting of nonlinear neurons was added, and in which a decoder provides the angle of reach movement instead of a vector (Text S1, Figure S2 and unpublished data). Note that in the framework of this more complex model, the noise can be unambiguously related to neuronal variability whereas in the simplified two-layer model considered in our paper, the noise is in the decoder.

One limitation of our work is that we did not model the trajectory of the movement and/or the muscle activation patterns needed to produce movements [43]. However, we expect that delayed learning and interferences also occur in a more detailed model of movement production, such as the one used in Legenstein et al. [34].

### Relation to previous works and predictions

A reward-based component in a sensorimotor task was shown to be involved in adaptation to rotations even when detailed spatial information regarding the error was provided to the subject [18], [19]. We investigated the ways in which neural possible mechanisms that reinforce successful actions affect adaptation dynamics. This type of reward-based mechanism was also studied in [17]. In this experiment, subjects adapted without visual feedback to a gradually increasing rotation of 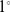 every 40 trials, up to an 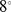 rotation. Our modeling results are in line with these experiments (Figure 4B). We thus predict that shaping the *reward* also accelerates adaptation.

Besides demonstrating that adaptation relying on rewards is possible by utilizing a gradual rotation paradigm, the Izawa and Shadmehr [17] results suggested that there is no change in the perceived sensory consequences of the motor commands; i.e., there should be no change in a “forward model”. Therefore, in [17] adaptation was modeled by an action selection rule. Our model is similar to the latter, as we focused on the reward-based component during adaptation. However, our model differs in that it is value-free, whereas in [17] it involved value-based reinforcement learning. Nevertheless, our model can also account for the experimental results reported in [17] for one target (see Text S1, Figure S3). Moreover, it allowed us to investigate the generalization curve and possible interference during adaptation for multiple targets.

#### The learning algorithm

Reward modulated learning rules have been used in previous modeling studies of sensorimotor tasks, such as birdsong acquisition [10] and motor learning in primates [34]. Similar rules have also been implemented in models of decision making [32], [35], [44] and association tasks [45]. The reward modulated rule we used here is a special case of REINFORCE learning rules. As shown by Williams [36], REINFORCE learning rules are equivalent on average to a gradient ascent algorithm on the average reward function. In fact, the gradient ascent dynamics with the average reward function (Eq.(10), averaged over the different movement directions) can be computed analytically. However, for finite 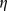 the actual trajectories can deviate substantially from the gradient ascent trajectory. In particular, delayed learning and the reduction in learning duration with the number of targets occurs for finite 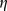 but these phenomena disappear when 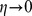 (unpublished data).

#### Shaping

Shaping strategies are used to teach subjects to perform operant conditioning tasks in a reasonable amount of time [22]. They were recently applied in the context of Reinforcement Learning by either increasing the complexity of the task [27], [46] or by shaping the reward function [26], [27], [47]. In the context of our model we showed that adaptation to one target can be accelerated if the target size or the rotation angle are progressively changed. This also reduces destructive interferences, thereby accelerating adaptation to multiple targets as well. We also showed that reward shaping can efficiently suppress destructive interferences and accelerates adaptation without compromising on performance.

To the best of our knowledge there are only a few theoretical works that have addressed shaping strategies in computational models in neuroscience (see e.g. [28]). Fiete et al. [10] used an adaptive threshold for reinforcement that adapts to performance. This is equivalent to the adaptive target size used here (Figure 4A). Smooth reward functions have been used in previous models of sensorimotor learning [34], [35], but the ways in which the shape of the reward function affects learning were not addressed.

#### Interference, delayed learning and generalization

The delayed learning effect exhibited by our network when it adapts to several targets is reminiscent of the slowing down that occurs in the model of birdsong learning in Fiete et al. [48]. In that model, a gradient ascent on a quadratic error function is performed by the network to learn a time dependent signal. The slowing down is due to destructive interferences in learning different temporal chunks of this signal. In fact, the presentation of multiple targets that involved a target in each trial, can be considered a discrete time dependent signal, and interferences when learning multiple targets can thus be seen as similar to interferences in different temporal chunks of the signal. However, in contrast to Fiete et al. [48], our network learns with a stochastic online learning rule, rather than a deterministic batch rule, and a different reward function is utilized.

Fiete et al. [48] suggested that to avoid interferences the avian brain exploits sparse neural representations. This solution is qualitatively similar to narrowing the tuning curves in our model. Similarly, Tanaka et al. [13] showed that narrow tuning curves can explain the independent learning of multiple targets in the context of a visuomotor rotation task with visual feedback. However, narrowing the tuning curves is not the only way to suppress destructive interferences, in that we showed here that they can also be suppressed by increasing the noise level, increasing the number of targets, and shaping the task or the reward.

Similarly to previous theoretical works on sensorimotor adaptation, we also showed that the shape of the generalization curve depends on the width of the tuning curves of the input neurons [4], [13], [14], [49]. In [17] it was shown that generalization in a reward-based rotation task falls to half of its maximum value already at 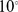 apart from the adapted direction. However, generalization above 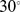 was not explored in this study. We therefore did not limit our model to a specific tuning width, as further experiments should be conducted to determine the generalization in the case of adaptation with rewards.

Negative generalization have been experimentally observed, both in adaptation to reaching movements under force-fields [4] and in visuomotor rotations with visual feedback [14]. In the latter study, the authors demonstrated that generalization curves are task-dependent, and showed how subjects negatively generalize the adaptation when targets that are far from the adapted target are presented. In fact, this study showed that generalization curves can even be non-monotonic. We predict here that this can also occur in the case of adaptation without sensory feedback.

As far as we can ascertain,, delayed learning in sensorimotor adaptation has not been reported before. For delayed learning to occur in our model, adaptation to one target needs to impair the performance on other targets and the reward must change abruptly around the target from a success to a failure. Under the assumptions we made, the shape of generalization curves can hint at on-line interferences that can be expected during adaptation. Therefore, because negative generalization was reported in a visuomotor adaptation task when the subject receives a continuous error [14], one might expect to find on-line interferences as well when visual feedback is available. However, in this case the error function does not change abruptly with respect to the distance to the target, as subjects are aware of the cursor location. Hence, when subjects receive visual feedback, we do not expect that interferences will result in substantial delayed learning or that learning will accelerate when the number of targets is large. We verified this expectation in the case of a quadratic error [13]. In particular, the learning duration increases monotonically with the number of targets and saturates when this number is large (Text S1; Figure S4).

On the other hand, in the case of adaptation with binary rewards, we do expect that if there are angles for which generalization is negative, delayed learning will be noticeable, as the reward function changes abruptly from a success to a failure (Figure 10).

### Conclusions and perspectives

The key finding of this theoretical work is that if a reward-modulated learning rule underlies adaptation, interferences are likely to be observed when learning multiple targets with a binary reward. It would be valuable to explore whether such effects occur in reward-based sensorimotor adaptation experiments with multiple sensory stimuli. We predict that for a binary reward function, destructive interferences will be observed if the neurons that encode the stimuli have broad tuning curves. These interferences are a dynamical counterpart of the generalization function and might result in a dramatic slowdown because of the abrupt change in the reward from success to failure around target size. We also predict that adding more targets should accelerate adaptation (Figure 11). From the learning curve of adaptation to one target, the rate and variability in which subjects adapt can be estimated. We predict that at parity of variability, subjects with larger learning rates will tend to display more destructive interferences and therefore slower adaptation to two targets (see Eq. (23)). By contrast,if the tuning curves are very narrow, destructive interferences are unlikely to be found. However, even in this case, when the stimuli are sufficiently close, constructive interferences should be observed. In this case as well, adding more targets should accelerate the adaptation.

Another prediction is that if adaptation is driven by reward modulated plasticity rules similar to the one we used here, smoothing the reward function should reduce interferences. In our model, this stems from the assumption of a reward modulated learning rule and not from the simplifying assumptions we made in constructing the model. Therefore, we suggest that testing this prediction could shed light on the synaptic mechanisms underlying adaptation tasks.

Finally, the location of the reward-based mechanism involved in adaptation could be the cortex-basal-ganglia network. As a matter of fact, there is evidence for the involvement of this network in pitch shift adaptation in songbirds. Although the neural correlates for adaptation in songbirds are unknown, when an auditory feedback is available to songbirds (by using miniature headphones [7]), the anterior frontal pathway, which is the avian homologue of the cortex-basal-ganglia network [50], is essential for adaptation based solely on binary rewards [15], [16]. Thus, exploring the behavioral and neural differences in auditory feedback versus binary reward adaptations in pitch shift experiments in songbirds may help reveal the neural mechanisms for reward-based adaptation.

## Materials and Methods

### The task

We consider a motor reaching task (see Figure 1A) in which a subject manually controls the location of a cursor on a screen to bring it within a circular target of radius 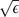 [16]. The target location is characterized by a two dimensional vector 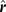 of norm 1 (we assume that the target is always at distance 1 from the center of the screen) and direction 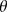. In a standard block of trials, the direction of motion of the cursor and the hand are the same. We assume that the subject is able to perform the task perfectly in this case. In a rotation block of trials a perturbation is introduced: the movement of the cursor on the screen is now rotated by an angle 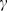 with respect to the hand movement. To overcome this perturbation the subject must move his hand in a direction 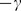 with respect to the target. Here we focus on the case where there is no visual feedback (the cursor is not on the screen): the only information the subject receives about his performance is provided by a reward signal delivered by the experimentalist [17].

### The network model

We consider a simplified computational model of a network performing this reaching task, see Figure 1B. The input layer of the network encodes the sensory information regarding the direction of the target, 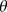. It is composed of 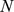 directionally tuned neurons labeled by their preferred direction, 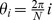 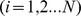. For simplicity, we assume that the shape of the tuning curves is the same for all neurons: upon presentation of a target in direction 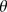 the activity of neuron 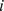 is 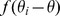. We take:
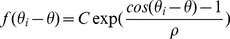
(2)where 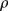 characterizes the width of the tuning curve and 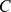 is the peak response of a neuron. The width of the tuning curves at half of its maximal activity relative to the baseline (half bandwidth) in this case is:
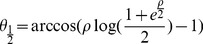
(3)

The second layer of the network encodes the location of the endpoint of the hand movement. It consists of two output units whose activities, 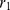 and 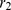, represent the two components of the hand position, 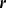. Upon presentation of a target in direction 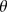:
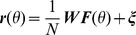
(4)where 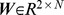 is the connectivity matrix between the two layers, 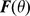 denotes the *N* dimensional vector of the input layer with components 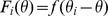, and 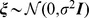 is a Gaussian noise. The location of the cursor at the end of the movement is related to 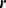 by a 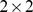 rotation matrix, 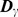, of angle 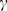. Therefore, the squared distance between the endpoint location of the cursor and the center of the target is:
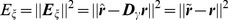
(5)where 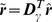. This quantity will be used to measure the error with which the network performs the reaching task. It is also useful to define the *noiseless* error:
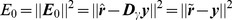
(6)where 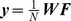. This quantity measures the error if the noise is suppressed.

Upon presentation of a target in a direction *θ* at trial *t*, the network performs the task and a reward *R* is delivered according to the outcome:
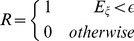
(7)

The matrix 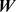 is then modified according to a reward-modulated learning rule:
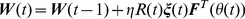
(8)where 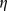 is the learning rate. This learning rule can be derived in a REINFORCE framework [36].

We assume that at the beginning of learning 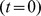, when there is no rotation, the network is able to perform the reaching task with zero noiseless error for all targets. When all the Fourier components of 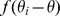 are non-zero, this constraint fully determines 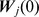:
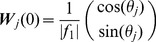
(9)where 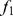 is the first Fourier component of the tuning curves. To get Eq. 10, one needs to calculate the Fourier expansion of 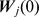 by using the constraint:
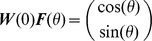
for each of the 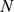 possible target directions, 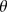. When some of the Fourier coefficients of the tuning curve function are zero, e.g. when using a cosine tuning curves, 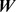 is determined up to the Fourier coefficient that are irrelevant to the above constraint. This does not affect the learning dynamics.

### Analysis of the model for adaptation to one target

#### Probability to generate a rewarded trial

The probability of generating a rewarded trial given the noiseless error at the end of the previous trial is:
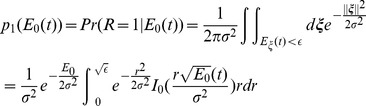
(10)where 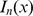 is the modified Bessel function of the first kind of order *n* [51]. The transition from the second to the third equation is done by a change of variables to polar coordinates, followed by the integration over the angle. Using this equation, we can calculate the probability to get the first reward in a given number of trials for an initial noiseless error, 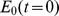. This probability is given by a geometrical distribution with a parameter 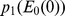 (defined as 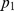 for simplicity). When 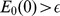, the expectation value of this distribution, 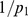, diverges for small values of 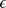 and 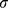.

#### Learning dynamics in the limit 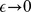


In the limit 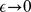, the probability of a trial to be rewarded decreases and thus the number of trials between rewarded trials diverges (see Eq. 10). However, one can still characterize the dynamics in terms of the evolution of the error as a function of the number of *rewarded* trials. The condition that the network generates the 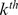 rewarded trial fully determines the noise:
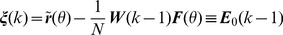
(11)The connectivity matrix is then updated according to:
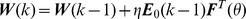

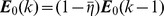
where the normalized learning rate is defined by:
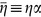
(12)with 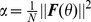. Solving the above recursion, one finds:
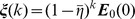

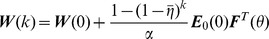


The error and the squared Frobenius norm of 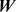 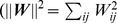) are then:
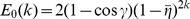

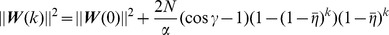
where we use the fact that 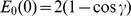.

The sequences 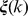, 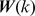 and 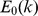 converge when 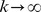 if:
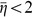
(13)Their limiting values are then:
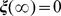

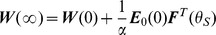

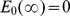

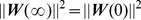
(14)Therefore, after enough rewarded trials the noiseless error goes down to zero. Note that there is no need to normalize the connectivity matrix after each update in this case, since in the large *k* limit the norm of the matrix returns to the value it had at 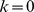.

#### The support of the noiseless error distribution is bounded

When 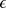 is finite, the noiseless error after a rewarded trial is:
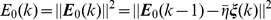
(15)where 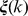 is such that the constraint in Eq. (7) holds, *i.e.*, 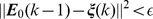. This constraint implies that 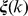 can be written as:
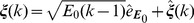
(16)where 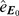 is the unit vector in the direction of 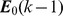 and 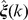 is a vector with a maximal norm 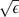. Inserting Eq. (16) into Eq. (15) one finds:
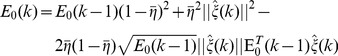
(17)

The noiseless error for a large number of trials is a random variable with a probability 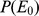 on the support 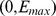. For vector 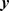 to be close to the target, the maximum value of the noiseless error, 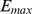, needs to be as small as possible. To estimate 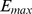, we compute the realization of 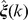 which maximizes the noiseless error, Eq. (18), at each rewarded trial *k*.

When 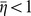, 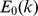 is maximal if 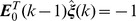 and 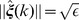. One then gets:
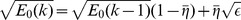
Solving the recursion gives:
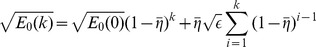
and therefore after a long time we get:
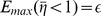
For 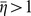, 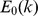 in Eq(17) is maximal if 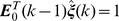 and 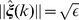. This leads to:
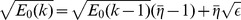
Solving the recursion and taking the limit 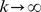, one gets that for 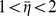:
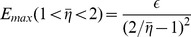
and when 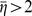:
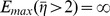
To summarize:
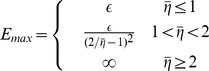
In particular, if 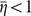 the noiseless error is guaranteed to always be smaller than 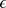 at large time.

#### Generalization error after adaptation to one target

Let us assume that the network has adapted to the rotation of the target presented in direction 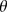. To measure the ability of the network to generalize to targets in other directions, we calculate the noiseless error for test target (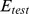), presented in a direction 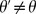 and define the generalization error by:
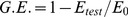
(18)

In the limit 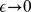 (assuming 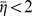), 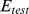 can be computed analytically, as function of 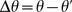. Using Eq. (15) one finds:
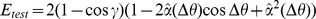
and
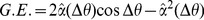
(19)where 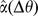 is:
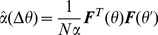
(20)depends on 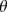 and 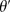 only via 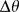. Note that 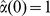. Specifically, in the limit of large 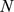 and when using the tuning curve function in Eq. (2), one gets:
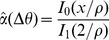
(21)where 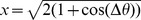.

### Adaptation to two targets

#### How does a reward affect the next trial?

Here we consider the case where the network adapts to two targets in the direction 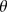 and 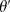 presented in alternation. If a rewarded trial occurs for one of the targets, the connectivity matrix is updated, affecting the noiseless error on the next trial when the other target is presented.

This noiseless error can be computed in the limit 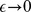. It is a good estimate for the noiseless error in the beginning of the adaptation with finite 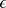, where the error is still big with respect to the target size. Let us assume that on trial *k* a target in direction *θ* is presented and that it is rewarded. This condition fully determines the realization of the noise on trial *k*, 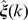. The noiseless errors for the two targets following the rewarded trial, denoted 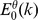 and 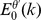, can be determined analytically. One finds:
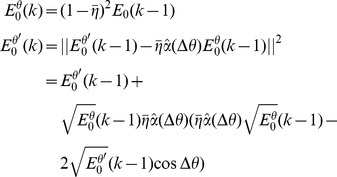
(22)

If 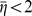, 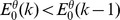, that is, the noiseless error for the target that has been rewarded decreases following the update of the connectivity matrix. For the other target (direction 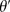), the effect of this update on the noiseless errors depends on the sign of the expression in parentheses in the second equation. If the two targets are in opposite directions, it is always positive and 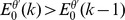. Thus, while the network performs better for one of the targets it performs worse for the other target. We term this situation *destructive* interference. On the other hand, if the targets are close such that the expression in parentheses is negative, 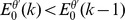. In other words, if the network improves for one of the targets it also improves for the other target. We term this situation *constructive* interference.

In particular, for the first rewarded trial, using 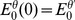
(0), we get:
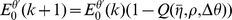
where:
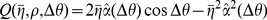
(23)We expect a constructive interference for 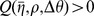 and destructive interferences otherwise. Note that for 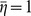 the interference function equals to the generalization error function (Eq. (20)). The transition between the constructive and destructive regimes is given by:
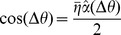
The quantity 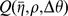) characterizes the strength of the interference. It can be a non-monotonous function of 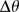. To show this, we calculate the derivative of 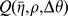 with respect to 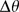, using Eq. (22). This derivative changes sign when:
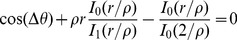
(24)For instance, when 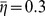 and 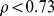 the function 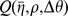 depends non-monotonically on 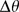. In other words, for sufficiently narrow tuning curves, the interference varies non-monotonically with the angular difference. However, this non-monotonicity effect can be very small: if the tuning curves are too narrow, 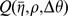 quickly reaches zero when increasing 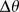.

### Numerical simulations

In the numerical simulations described in this paper, the input layer consists of 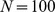 neurons. We normalized the tuning curves (parameter 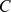 in Eq. (2)) such that 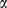 remains constant (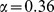) when changing 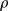. This was done to guarantee that the time to learn one target does not depend on the tuning width.

#### Learning duration and final error

We define the final error of the network as the median of the error over the last 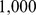 trials of the simulation for each realization. We then determine the learning duration, 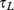, as the trial number at which the filtered signal (median filter with a window length of 50 trials) crosses a threshold, defined to be 5% above the final error. In order to avoid boundary problems of the filter at time 0 (the discontinuity in the error when we induce the rotation), we calculate the error at 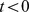 while assuming that the cursor is already rotated (even though it did not). In the figures we plot the actual error before the rotation, which is small. Similar results were obtained using a linear filter.

#### Time dependent correlations between the errors for two targets

When the network adapts to a rotation for two targets presented in alternation in consecutive trials, the learning processes for the two targets interfere. This interference can be quantified by considering the correlations between the errors on two consecutive trials:
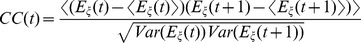
The brackets denote the average over repetitions of the adaptation process, which differ by the realization of the noise. Negative 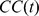 indicates that if the network improves for one target it deteriorates for the other target (destructive interference). Positive 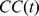 corresponds to constructive interference.

#### Performance and noiseless performance

We ran long simulations of 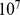 trials to estimate the performance and noiseless performance after the transient learning phase. The performance is given by:
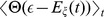
(25)and the noiseless performance is given by:
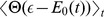
(26)where 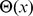 is the Heaviside function and the average is over time, when the transient learning phase was excluded.

## Supporting Information

Figure S1Delayed learning effect with an on-line gradient ascent algorithm. **A.** Delayed learning in a reward function that varies abruptly with the error (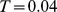). **B.** The delayed learning is reduced for a smoother reward function. (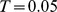). **C.** The delayed learning almost disappears when the reward function is smoothed even further (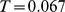). Note the change of scale in the abscissa. Parameters: 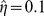, 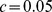, 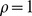.(EPS)

Figure S2Delayed learning effect in a 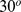 rotation for two targets in the 3-layer network. The reach angle (in degrees) is plotted as a function of the trial number. The shaded area corresponds to the target size. Initial conditions as explained in the text. Parameters: 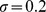, 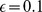, 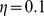, 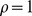.(EPS)

Figure S3Gradual adaptation for an 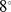 rotation. **A.** Reach angle (in degrees) as a function of the trial number when the rotation angle is increased by 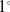 every 40 trials up to 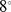. The shaded area corresponds to the target size (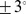 around the target center). 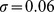. **B.** The generalization error, given as the change in reach angle. The learned target is at 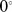. Circles : simulation results. For clarity, the results are displayed for test targets sampled every 2.5 degrees. Solid line: analytical results. Shaded area corresponds to the standard deviation in generalization error in numerical simulations estimated over 100 repetitions. Number of neurons in the input layer: 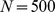. **C.** The shape of the tuning curves that was used in (**A**) and (**B**): 
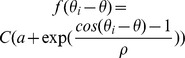
, where 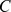 is a normalization constant (see Materials and Methods), 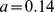, 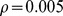.(EPS)

Figure S4Learning duration when adapting to multiple targets varies monotonically with the number of learned targets when using a gradient descent on a quadratic error function. Total number of target presentations required to learn the entire task *vs.* the number of presented targets, *m*. The targets are evenly distributed (between 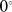 to 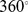). Learning duration was calculated as the trial number at which learning curve crossed a threshold of 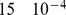. Color coded as in Figure 11 in the Results. Black: 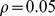. Blue: 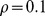. Purple: 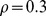. Green: 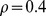. Dashed black line corresponds to learning the targets independently. Compare with Figure 11 in main text.(EPS)

Text S1 This document is a supporting text for the supplementary figures.
(PDF)

## References

[1] Pouget A, Snyder L (2000) Computational approaches to sensorimotor transformations. Nature Neuroscience 3: 1192–1198. 11127837 10.1038/81469

[2] Piaget J, Cook M (1953) The origin of intelligence in the child. London: Routledge & Kegan Paul.

[3] Krakauer J, Pine Z, Ghilardi M, Ghez C (2000) Learning of visuomotor transformations for vectorial planning of reaching trajectories. The Journal of Neuroscience 20: 8916–8924. 11102502 10.1523/JNEUROSCI.20-23-08916.2000 PMC6773094

[4] Thoroughman K, Shadmehr R (2000) Learning of action through adaptive combination of motor primitives. Nature 407: 742–747. 11048720 10.1038/35037588 PMC2556237

[5] Chou I, Lisberger S, et al. (2002) Spatial generalization of learning in smooth pursuit eye movements: implications for the coordinate frame and sites of learning. The Journal of Neuroscience 22: 4728–4739. 12040080 10.1523/JNEUROSCI.22-11-04728.2002 PMC2548309

[6] Linkenhoker B, Knudsen E (2002) Incremental training increases the plasticity of the auditory space map in adult barn owls. Nature 419: 293–296. 12239566 10.1038/nature01002

[7] Sober S, Brainard M (2009) Adult birdsong is actively maintained by error correction. Nature neuroscience 12: 927–931. 19525945 10.1038/nn.2336 PMC2701972

[8] Houde J, Jordan M (1998) Sensorimotor adaptation in speech production. Science 279: 1213–1216. 9469813 10.1126/science.279.5354.1213

[9] Sober S, Brainard M (2012) Vocal learning is constrained by the statistics of sensorimotor experience. Proceedings of the National Academy of Sciences 109: 21099–21103. 10.1073/pnas.1213622109 PMC3529072 23213223

[10] Fiete I, Fee M, Seung H (2007) Model of birdsong learning based on gradient estimation by dynamic perturbation of neural conductances. Journal of Neurophysiology 98: 2038–2057. 17652414 10.1152/jn.01311.2006

[11] Rokni U, Richardson A, Bizzi E, Seung H (2007) Motor learning with unstable neural representations. Neuron 54: 653–666. 17521576 10.1016/j.neuron.2007.04.030

[12] Poggio T, Bizzi E (2004) Generalization in vision and motor control. Nature 431: 768–774. 15483597 10.1038/nature03014

[13] Tanaka H, Sejnowski T, Krakauer J (2009) Adaptation to visuomotor rotation through interaction between posterior parietal and motor cortical areas. Journal of Neurophysiology 102: 2921–2932. 19741098 10.1152/jn.90834.2008 PMC2777823

[14] Taylor J, Hieber L, Ivry R (2013) Feedback-dependent generalization. Journal of Neurophysiology 109: 202–215. 23054603 10.1152/jn.00247.2012 PMC3545161

[15] Tumer E, Brainard M (2007) Performance variability enables adaptive plasticity of crystallized adult birdsong. Nature 450: 1240–1244. 18097411 10.1038/nature06390

[16] Andalman A, Fee M (2009) A basal ganglia-forebrain circuit in the songbird biases motor output to avoid vocal errors. Proceedings of the National Academy of Sciences 106: 12518–12523. 10.1073/pnas.0903214106 PMC2709669 19597157

[17] Izawa J, Shadmehr R (2011) Learning from sensory and reward prediction errors during motor adaptation. PLoS computational biology 7: e1002012. 21423711 10.1371/journal.pcbi.1002012 PMC3053313

[18] Shmuelof L, Huang V, Haith A, Delnicki R, Mazzoni P, et al. (2012) Overcoming motor forgetting through reinforcement of learned actions. The Journal of Neuroscience 32: 14617–14621. 23077047 10.1523/JNEUROSCI.2184-12.2012 PMC3525880

[19] Huang V, Haith A, Mazzoni P, Krakauer J (2011) Rethinking motor learning and savings in adaptation paradigms: model-free memory for successful actions combines with internal models. Neuron 70: 787–801. 21609832 10.1016/j.neuron.2011.04.012 PMC3134523

[20] Paz R, Boraud T, Natan C, Bergman H, Vaadia E (2003) Preparatory activity in motor cortex reflects learning of local visuomotor skills. Nature Neuroscience 6: 882–890. 12872127 10.1038/nn1097

[21] Warren T, Tumer E, Charlesworth J, Brainard M (2011) Mechanisms and time course of vocal learning and consolidation in the adult songbird. Journal of Neurophysiology 106: 1806–1821. 21734110 10.1152/jn.00311.2011 PMC3191835

[22] Skinner B (1967) Science and human behavior. Free Press.

[23] Lawrence D (1952) The transfer of a discrimination along a continuum. Journal of Comparative and Physiological Psychology 45: 511. 13000022 10.1037/h0057135

[24] Terrace H (1963) Discrimination learning with and without errors. Journal of the Experimental Analysis of Behavior 6: 1. 13980667 10.1901/jeab.1963.6-1 PMC1404228

[25] Kangas B, Bergman J (2012) A novel touch-sensitive apparatus for behavioral studies in unrestrained squirrel monkeys. Journal of Neuroscience Methods 209: 331–6. 22790109 10.1016/j.jneumeth.2012.06.028 PMC3429786

[26] Ng A, Harada D, Russell S (1999) Policy invariance under reward transformations: Theory and application to reward shaping. In: Machine learning: proceedings of the Sixteenth International Conference (ICML'99). Morgan Kaufmann Pub, p. 278.

[27] Randløv J (2000) Shaping in reinforcement learning by changing the physics of the problem. In: Proc. 17th International Conf. on Machine Learning. pp. 767–774.

[28] Krueger K, Dayan P (2009) Flexible shaping: How learning in small steps helps. Cognition 110: 380–394. 19121518 10.1016/j.cognition.2008.11.014

[29] Kerr J, Wickens J (2001) Dopamine d-1/d-5 receptor activation is required for long-term potentiation in the rat neostriatum in vitro. Journal of Neurophysiology 85: 117–124. 11152712 10.1152/jn.2001.85.1.117

[30] Ding L, Perkel D (2004) Long-term potentiation in an avian basal ganglia nucleus essential for vocal learning. The Journal of Neuroscience 24: 488–494. 14724247 10.1523/JNEUROSCI.4358-03.2004 PMC6729982

[31] Reynolds J, Hyland B, Wickens J (2001) A cellular mechanism of reward-related learning. Nature 413: 67–70. 11544526 10.1038/35092560

[32] Loewenstein Y, Seung H (2006) Operant matching is a generic outcome of synaptic plasticity based on the covariance between reward and neural activity. Proceedings of the National Academy of Sciences 103: 15224–15229. 10.1073/pnas.0505220103 PMC1622804 17008410

[33] Reynolds JN, Wickens JR (2002) Dopamine-dependent plasticity of corticostriatal synapses. Neural Networks 15: 507–521. 12371508 10.1016/s0893-6080(02)00045-x

[34] Legenstein R, Chase S, Schwartz A, Maass W (2010) A reward-modulated hebbian learning rule can explain experimentally observed network reorganization in a brain control task. The Journal of Neuroscience 30: 8400–8410. 20573887 10.1523/JNEUROSCI.4284-09.2010 PMC2917246

[35] Frémaux N, Sprekeler H, Gerstner W (2010) Functional requirements for reward-modulated spike-timing-dependent plasticity. The Journal of Neuroscience 30: 13326–13337. 20926659 10.1523/JNEUROSCI.6249-09.2010 PMC6634722

[36] Williams R (1992) Simple statistical gradient-following algorithms for connectionist reinforcement learning. Machine learning 8: 229–256.

[37] Fiete I, Seung H (2006) Gradient learning in spiking neural networks by dynamic perturbation of conductances. Physical review letters 97: 48104. 10.1103/PhysRevLett.97.048104 16907616

[38] Werfel J, Xie X, Seung H (2005) Learning curves for stochastic gradient descent in linear feedforward networks. Neural computation 17: 2699–2718. 16212768 10.1162/089976605774320539

[39] Mandelblat-Cerf Y, Paz R, Vaadia E (2009) Trial-to-trial variability of single cells in motor cortices is dynamically modified during visuomotor adaptation. The Journal of Neuroscience 29: 15053–15062. 19955356 10.1523/JNEUROSCI.3011-09.2009 PMC6665974

[40] Ölveczky B, Otchy T, Goldberg J, Aronov D, Fee M (2011) Changes in the neural control of a complex motor sequence during learning. Journal of Neurophysiology 106: 386–397. 21543758 10.1152/jn.00018.2011 PMC3129720

[41] Körding K, Wolpert D (2004) The loss function of sensorimotor learning. Proceedings of the National Academy of Sciences of the United States of America 101: 9839–9842. 15210973 10.1073/pnas.0308394101 PMC470761

[42] Schultz W (1998) Predictive reward signal of dopamine neurons. Journal of neurophysiology 80: 1–27. 9658025 10.1152/jn.1998.80.1.1

[43] Izawa J, Kondo T, Ito K (2004) Biological arm motion through reinforcement learning. Biological cybernetics 91: 10–22. 15309543 10.1007/s00422-004-0485-3

[44] Law C, Gold J (2009) Reinforcement learning can account for associative and perceptual learning on a visual-decision task. Nature Neuroscience 12: 655–663. 19377473 10.1038/nn.2304 PMC2674144

[45] Vladimirskiy B, Vasilaki E, Urbanczik R, Senn W (2009) Stimulus sampling as an exploration mechanism for fast reinforcement learning. Biological cybernetics 100: 319–330. 19360435 10.1007/s00422-009-0305-x

[46] Taylor M, Stone P (2009) Transfer learning for reinforcement learning domains: A survey. The Journal of Machine Learning Research 10: 1633–1685.

[47] Laud A, DeJong G (2003) The influence of reward on the speed of reinforcement learning: An analysis of shaping. In: Proc. 12th International Conf. on Machine Learning (ICML-2003). volume 20, p. 440.

[48] Fiete I, Hahnloser R, Fee M, Seung H (2004) Temporal sparseness of the premotor drive is important for rapid learning in a neural network model of birdsong. Journal of Neurophysiology 92: 2274–2282. 15071087 10.1152/jn.01133.2003

[49] Donchin O, Francis J, Shadmehr R (2003) Quantifying generalization from trial-by-trial behavior of adaptive systems that learn with basis functions: theory and experiments in human motor control. The Journal of neuroscience 23: 9032–9045. 14534237 10.1523/JNEUROSCI.23-27-09032.2003 PMC6740843

[50] Gale S, Perkel D (2010) Anatomy of a songbird basal ganglia circuit essential for vocal learning and plasticity. Journal of chemical neuroanatomy 39: 124–131. 19596062 10.1016/j.jchemneu.2009.07.003 PMC2822067

[51] Gradshtei˘n I, Ryzhik I, Jeffrey A, Zwillinger D (2007) Table of integrals, series, and products. Elsevier academic press.

