# Supplementary material for: Interference and Shaping in Sensorimotor Adaptations with Rewards
Source: PLoS Comput Biol. 2014 Jan 9;10(1):e1003377. doi: 10.1371/journal.pcbi.1003377 (PMC3886885; doi:10.1371/journal.pcbi.1003377)
Supplement: Text S1 — This document is a supporting text for the supplementary figures. (PDF) [file pcbi.1003377.s005.pdf]

Text S1

Delayed learning for a Gradient Ascent over a sigmoidal reward function

The delayed learning effect described in the main text depends mainly on the width of the tuning curves and on the shape of the reward, which changes abruptly from zero to one. This was shown in the context of a learning rule derived from the REINFORCE family. In fact, delayed learning is not limited to this type of algorithm. It also occurs if learning is driven by an on-line gradient ascent on a sigmoidal reward function according to the rule:

$$\boldsymbol{W}(t) = \boldsymbol{W}(t-1) + 2\hat{\eta}\frac{\partial R(E(t))}{\partial E}\boldsymbol{E}(t)\mathbf{F}^T(\theta(t))$$

where E and $\boldsymbol{E}$ are defined in Eq.(6) in the main text. Note that this learning rule is deterministic.

Figure S1 displays the results for the sigmoidal reward function:

$$R(E) = \frac{1}{1 + \exp{(E+c)/T}}$$

with a constant c and a smoothing parameter T (see Results). Here, as in Figure 10B in the main text, delayed learning is reduced when the reward function is smoothed.

Delayed learning for a network with an intermediate layer and a different decoder

We consider here a network with three layers. It consists of an N dimensional sensory input layer and a 2 dimensional output layer, as in the model investigated in the main text. In addition, it has an N dimensional hidden layer.

The activity of the neurons in the hidden layer is:

$$\boldsymbol{X} = \boldsymbol{W}\boldsymbol{F}(\theta) + \boldsymbol{\xi}$$

where $\boldsymbol{\xi} \sim \mathcal{N}(0, \sigma^2 \boldsymbol{I})$ is a Gaussian noise. The direction of the reach movement is then computed as the angle of the vector:

$$\boldsymbol{r} = \frac{1}{N}\boldsymbol{D}\boldsymbol{W}\boldsymbol{F}(\theta) + \frac{1}{\sqrt{N}}\boldsymbol{D}\boldsymbol{\xi}$$

with:

$$\boldsymbol{D}_j = \begin{pmatrix} \cos(\theta_j) \\ \sin(\theta_j) \end{pmatrix}$$

The $\frac{1}{\sqrt{N}}$ normalization factor guarantees that the variability of $\boldsymbol{r}$ does not depend on N. When a rotation is introduced, an angle γ is added to the decoded direction, denoted by θ_r. The error is then calculated according to:

$$E_\xi = (\cos(\theta_r + \gamma) - \cos(\theta))^2 + (\sin(\theta_r + \gamma) - \sin(\theta))^2$$

where θ is the location of the target . We stick with the notation E_ξ to highlight the fact that this measure depends on the noise through θ_r. This quantity will be used to measure the error with which the network performs the reaching task.

Upon presentation of a target in a direction θ at trial t, the network performs the task and a reward R is delivered according to the outcome:

$$R = \begin{cases} 1 & E_\xi < \epsilon \\ 0 & otherwise \end{cases}$$

The matrix $\boldsymbol{W}$ is then updated in two steps:

$$\boldsymbol{W}(t) = \boldsymbol{W}(t-1) + \eta R(t)\boldsymbol{\xi}(t)\mathbf{F}^T(\theta(t))$$

$$\boldsymbol{W} = ||\boldsymbol{r}||^{-1}\boldsymbol{W}$$

The second step prevents a drift of the weight matrix in directions that are irrelevant to the decoder of the angle.

We first train the network to perform the reaching task without a rotation on 15 targets, with a small error for all targets. The adaptation to the rotation is then performed with this initial condition. Figure S2 plots the reach angle against the trial number in a network adapting to two targets to a rotation of 30°. Although the network adapts to the rotation for the target at $\theta = 0°$ quite fast, adaptation to the target in the opposite direction ($\theta = 180°$) is delayed. Similar results can be achieved as well with threshold-linear neurons in the intermediate layer (unpublished data).

Generalization error for gradual adaptation

Here we compare the behavior of our model for gradual adaptation to one target with the corresponding experimental and modeling results reported in [1]. As shown in Figure S3, our model accounts for the experimental data to the same extent as the model studied in the latter paper (compare with Figure 1C and 2B in [1]). We also found that in our model, for a large rotation (e.g. 30 degrees), there is bias toward the reinforced location, but this bias is negligible for a small rotation angle (e.g. 8 degrees, as in [1]).

Learning the rotation for multiple targets with minimization of a quadratic error

The increase in the learning duration for wide tuning curves in our model, as well as the decrease in learning duration when multiple targets are learned, stem from the shape of the reward. These effects do not occur if the adaptation is done using an on-line gradient descent on a quadratic error function, as for instance in the model explored in [2]. This is shown in Figure S4.

References

1. Izawa J, Shadmehr R (2011) Learning from sensory and reward prediction errors during motor adaptation. PLoS computational biology 7: e1002012.

2. Tanaka H, Sejnowski T, Krakauer J (2009) Adaptation to visuomotor rotation through interaction between posterior parietal and motor cortical areas. Journal of Neurophysiology 102: 2921–2932.
